# The Role of Hydrogen Sulfide in Regulation of Cell Death following Neurotrauma and Related Neurodegenerative and Psychiatric Diseases

**DOI:** 10.3390/ijms241310742

**Published:** 2023-06-28

**Authors:** Stanislav Rodkin, Chizaram Nwosu, Alexander Sannikov, Margarita Raevskaya, Alexander Tushev, Inna Vasilieva, Mitkhat Gasanov

**Affiliations:** 1 Department of Bioengineering, Faculty of Bioengineering and Veterinary Medicine, Don State Technical University, 344000 Rostov-on-Don, Russia; 2 Department of Psychiatry, Rostov State Medical University, 344022 Rostov-on-Don, Russia; 3 Neurosurgical Department, Rostov State Medical University Clinic, 344022 Rostov-on-Don, Russia; 4 N.V. Sklifosovsky Institute of Clinical Medicine, Department of Polyclinic Therapy, I.M. Sechenov First Moscow State Medical University, 119435 Moscow, Russia; 5 Department of Internal Diseases #1, Rostov State Medical University, 344022 Rostov-on-Don, Russia

**Keywords:** hydrogen sulfide, neurotrauma, apoptosis, autophagy, ferroptosis, pyroptosis, neuron, glial cells, cognitive impairment, encephalopathy, depression, anxiety disorders, epilepsy, neurodegenerative diseases

## Abstract

Injuries of the central (CNS) and peripheral nervous system (PNS) are a serious problem of the modern healthcare system. The situation is complicated by the lack of clinically effective neuroprotective drugs that can protect damaged neurons and glial cells from death. In addition, people who have undergone neurotrauma often develop mental disorders and neurodegenerative diseases that worsen the quality of life up to severe disability and death. Hydrogen sulfide (H_2_S) is a gaseous signaling molecule that performs various cellular functions in normal and pathological conditions. However, the role of H_2_S in neurotrauma and mental disorders remains unexplored and sometimes controversial. In this large-scale review study, we examined the various biological effects of H_2_S associated with survival and cell death in trauma to the brain, spinal cord, and PNS, and the signaling mechanisms underlying the pathogenesis of mental illnesses, such as cognitive impairment, encephalopathy, depression and anxiety disorders, epilepsy and chronic pain. We also studied the role of H_2_S in the pathogenesis of neurodegenerative diseases: Alzheimer’s disease (AD) and Parkinson’s disease (PD). In addition, we reviewed the current state of the art study of H_2_S donors as neuroprotectors and the possibility of their therapeutic uses in medicine. Our study showed that H_2_S has great neuroprotective potential. H_2_S reduces oxidative stress, lipid peroxidation, and neuroinflammation; inhibits processes associated with apoptosis, autophagy, ferroptosis and pyroptosis; prevents the destruction of the blood-brain barrier; increases the expression of neurotrophic factors; and models the activity of Ca^2+^ channels in neurotrauma. In addition, H_2_S activates neuroprotective signaling pathways in psychiatric and neurodegenerative diseases. However, high levels of H_2_S can cause cytotoxic effects. Thus, the development of H_2_S-associated neuroprotectors seems to be especially relevant. However, so far, all H_2_S modulators are at the stage of preclinical trials. Nevertheless, many of them show a high neuroprotective effect in various animal models of neurotrauma and related disorders. Despite the fact that our review is very extensive and detailed, it is well structured right down to the conclusions, which will allow researchers to quickly find the proper information they are interested in.

## 1. Introduction

Neurotrauma is one of the leading causes of disability and death worldwide. It takes third position after cardiovascular and oncological diseases. Moreover, due to neurotrauma, the highest mortality and proportion of disability is observed among the young population. This situation is complicated due to the lack of effective clinical neuroprotective drugs that can protect neurons and glial cells from traumatic injury [1,2]. In addition, the heterogeneity of neurotrauma creates additional difficulties in their study and difficulties in developing and selecting a competent treatment strategy [3]. In addition, CNS and PNS injuries often lead to various mental disorders [4,5,6,7,8] and neurodegenerative diseases [9,10,11,12,13], which are accompanied by increased cell death of the nervous tissue [14,15,16,17,18,19]. It is worth noting that nerve cells are very sensitive to various influences, including microwave radiation, which can cause neurodegenerative diseases [20]. This confirms the presence of complex intermolecular interactions in the nervous tissue. To solve these problems, it is necessary to search for promising molecular targets and study the intracellular signaling processes associated with them [21].

Gasotransmitters are important signaling gaseous molecules that perform various functions in the body under normal and pathological conditions [22]. They play an important role in the processes of cell survival and death [23]. Although many signaling mechanisms of cytoprotection and cytotoxicity of these messengers are poorly understood and often contradictory, the S-gasotransmitter H_2_S remains of great interest to researchers, especially in conditions of traumatic damage to the nervous system [24,25,26], and mental [27] and neurodegenerative diseases [28].

H_2_S is produced endogenously in many tissues and is involved in various cellular processes: neurotransmission, apoptosis, inflammation, oxidative stress, angiogenesis, etc. [22]. Many scientific data indicate that H_2_S can act both as a neuroprotective agent and as a factor responsible for neurodegeneration [25,29,30,31]. Its role in neurotrauma is also ambiguous: some researchers point to its pronounced neuroprotective effect [29,32,33,34,35,36], while others associate it with cell death [37,38,39,40]. Of particular interest are the H_2_S-dependent signaling mechanisms of survival and death of nerve cells in mental disorders [4] and neurodegenerative diseases [41], which often develop with neurotrauma [4,5,6,7,8,9,10,11,12,13].

Thus, the purpose of this review is a comprehensive analysis of the current data on the role of H_2_S in the survival and death of neurons and glial cells in neurotrauma and related mental disorders and neurodegenerative diseases. This large-scale review study is a natural continuation of a recently published review in which we summarized scientific research on the role of gasotransmitters in apoptotic cell death in cardiovascular, renal, rheumatic, neurodegenerative diseases and mental disorders [42]. As a result, we decided to focus our attention on H_2_S as a potential cytoprotective signaling molecule. In this global review, the role of H_2_S-dependent signaling mechanisms in the survival and death of neurons and glial cells in injuries of the brain, spinal cord and PNS is comprehensively considered in detail and the signaling pathways underlying the pathogen of neurodegenerative diseases and mental disorders associated with neurotrauma are studied.

## 2. Materials and Methods

This large review study was performed in accordance with the Preferred Reporting Items for Systematic Reviews and Meta-Analyses Extension for Scoping Reviews (PRISMA-ScR) [43]. The Technical Expert Group (TEG) consisted of 7 highly qualified specialists, including 3 molecular biologists (S.R., C.N., M.R.) and 4 clinical and psychiatric physicians (A.S., A.T., I.V., M.G.).

TEG sought to investigate the role of H_2_S in cell death in neurotrauma and related neurodegenerative and psychiatric illnesses. TEG searched for studies, assessed their reliability, and conceptualized and synthesized the findings.

### 2.1. Finding Sources

TEG searched for sources in PubMed (Public MedLine, managed by the National Center of Biotechnology Information (NCBI) of the National Library of Medicine in Bethesda (Bethesda, MD, USA)), as well as in Web of Science, and Scopus. Our strategy search did not include restrictions on publication date, language and free full-text access in order to obtain the maximum coverage for the given parameters, namely keywords. In addition, to improve search efficiency, synonyms and terms associated with keywords and their phrases were used. We used logical operators such as “AND”, “OR” next to the keywords to create a logical string that allows us to refine our search and find the most relevant sources (Table 1). Additionally, we could use the logical operator “NOT” [44].

### 2.2. Study Quality Assessment

At this stage, three authors (M.R., A.T., A.S.) independently checked the titles/abstracts of the sources that were obtained from the databases. In the case where one author recognized an article suitable for inclusion in a system analysis, the other authors analyzed it in detail. In addition, this process was controlled, and the first author (S.R.) acted as an independent arbiter. In case of a disagreement between the authors, the final decision on the inclusion or exclusion of an article was made by the first (S.R.) or the last author (M.G.).

The studies that were included in our systematic review had to meet the following criteria: (1) transparency of the results obtained; (2) competent statistical data analysis; (3) representative sample; competent choice of materials and methods for research; (4) correct interpretation of the data.

### 2.3. Conceptualization and Synthesis of the Received Data

The data obtained were subjected to detailed analysis by the entire TEG group. The obtained data were conceptualized and synthesized in the form of textual and graphic representation. The final evaluation of the results obtained was carried out by the first author (S.R.).

## 3. Classification and Molecular Mechanisms of Neurotrauma

Neurotrauma is damage to various structures of the CNS and PNS caused by external forces. It includes isolated and combined traumatic brain injury (TBI); isolated and combined spinal cord injury (SCI); and multiple limb injury with isolated or combined damage to bones, ligaments, blood vessels, and peripheral nerves [45]. Currently, it is known that neurotrauma can lead to various mental and neurodegenerative diseases. This can be based on both molecular mechanisms and direct mechanical damage to anatomically important structures of the CNS and PNS. However, before elucidating this issue in more detail, we will consider, in general, the classification of neurotrauma and the main molecular mechanisms that accompany this pathological condition.

TBI and SCI in young and middle-aged men is ahead of cardiovascular and oncological diseases. Along with this, injuries of the PNS are a major public health problem [1,2,46]. With mechanical damage in the nervous tissue, various pathological processes develop, leading to the death of neurons. The treatment of these neuropathological processes is a major public health problem worldwide. However, effective clinical neuroprotective drugs have not yet been found. Their search requires deep and comprehensive studies of the molecular mechanisms of neurodegeneration and neuroprotection in these pathological processes [2].

TBI is a type of damage where the skull suffers from mechanical effects, as well as intracranial formations—the brain, meninges, blood vessels, cranial nerves. TBI is a heterogeneous pathological condition [25,47,48,49]. The destruction of nervous tissue in TBI is due to primary and secondary mechanisms of brain damage. Primary damage is caused by the direct impact of mechanical energy on the substance of the brain. In the area of primary brain damage, necrosis of brain tissue, death of neurons and glial cells, axonal ruptures and vascular thrombosis occur [25,47,48,49,50]. As a result, a focus is formed, protected by a penumbra—a zone of moderate ischemia. Cell death in the penumbra region leads to the expansion of the zone of the necrotic focus of TBI [51,52].

Secondary brain damage develops in response to primary mechanical damage, which triggers a cascade of molecular cellular events: oxidative phosphorylation in the mitochondria is disrupted, intracellular Ca^2+^ concentration increases, free oxygen radicals and vasoactive metabolites of arachidonic acid are released, the mechanisms of the complement cascade and lipid peroxidation are activated, and so on [53]. A sharp activation of the metabolic processes in neurons leads to ATP pool depletion and a disruption of the functions of Ca^2+^ channels. As a result, there is an increase in the permeability of the cell membranes to Ca^2+^ ions and the release of Ca^2+^ from intracellular depots, which leads to the depolarization of neurons and the release of glutamate, which activates N-methyl-D-aspartic acid (NMDA) receptors (NMDARs). Intracellular overload of Ca^2+^ occurs, which triggers a whole cascade of reactions associated with the activation of phospholipases, proteases and nucleases, the lysis of structural proteins, the expression of pro-apoptotic genes, the release of cell death factors from mitochondria, hyper synthesis of nitric oxide, and oxidative stress [54,55]. Hence, leading to the apoptotic death of neurons and glial cells [47].

Another major traumatic injury to the central nervous system is SCI. This is characterized by compression, or partial or complete rupture of the spinal cord. This group of neurotraumas is characterized by high disability and mortality and is practically untreatable. [56,57]. Spinal cord injury can be characterized by the destruction of several, many, or all of the nerve fibers that pass through the injury site. Recovery after this type of neurotrauma is complicated by the extremely weak regenerative capabilities of the spinal cord and is usually possible only with mild damage; with slight death of nerve cells and slight destruction of spinal nerve fibers [58]. This type of injury is accompanied by primary and secondary injuries similar to TBI [59].

Furthermore, injuries to the PNS are of great danger, and often lead to a deterioration in the quality of life up to severe disability or death [60]. Peripheral nerve damage is the result of the destruction of nerves that extend from the spinal cord and brain to various parts of the body and are located outside of the CNS [61]. These nerves can be damaged as a result of various factors, such as trauma, disease, inflammation, etc. Of particular danger are injuries to peripheral nerves, often accompanied by their complete rupture, that is, axotomy (AT), which initiates a complex cascade of signaling and metabolic processes aimed at the death or survival of the neuron [62,63].

AT is characterized by three main molecular-cellular events: Wallerian degradation of the severed axon, death of the damaged neuron, or its regeneration with the regrowth of the axon and the restoration of nerve connections. The peculiarity of PNS neurons is their ability to regenerate a damaged axon, while CNS neurons degenerate and die as a result of AT [64]. About 30% of PNS motor and sensory neurons survive AT by restoring nerve connections. An important factor associated with the survival of nerve cells in AT is the distance from the site of the axon rupture to the soma. Generally, the larger it is, the higher the chances of neuron regeneration [65,66].

H_2_S plays an important role in pathological conditions and may also be involved in processes associated with inflammation [67,68], oxidative stress synthesis [32,34,69], apoptosis [39,40], and autophagy [25,50], etc.

## 4. The Role of Neurotrauma in the Pathogenesis of Mental Disorders

Neurotrauma of various origins can lead to cognitive impairment [4,5,6,7,8], depression and anxiety disorders [70,71,72], epilepsy [73,74,75,76], encephalopathy [77,78], neurodegenerative disorders [9,10,11,12,13] and chronic pain [79]. Moreover, all these pathological conditions can be accompanied by increased cell death, in particular, apoptosis [14,15,16,17,18,19], neuroinflammation [80,81,82,83], and oxidative stress [84,85,86,87], etc.

### 4.1. Traumatic Brain Injury

TBI often causes cognitive impairment [4,5], encephalopathy [77,78], depression and anxiety disorders [70,71], epilepsy [73,74], chronic pain [79], and increases the risk of developing neurodegenerative diseases [9,10].

For example, TBI can cause cognitive impairment as a result of damage to hippocampal neurons, which are especially vulnerable to trauma, and changes in the synaptic plasticity in this area. Impaired neurotransmission induced by activation of Ca^2+^-dependent phosphatases and proteases, loss of dendritic spines, Ca^2+^-excitotoxicity, increased levels of apoptosis, autophagy, necroptosis, and NMDAR activation, are also a key factor in the development of cognitive impairment in TBI [88].

TBI-induced depression may be the result of damage to the frontal lobe, white matter, amygdala, and disruption of the neuronal network between different areas of the brain [70]. Bruises and swelling of the brain, intracranial hematomas, and changes in the blood-brain barrier, are concomitant negative factors in TBI, which often lead to epileptic seizures [2]. The dysfunction of downstream inhibitory regulation, loss of neurons in noradrenergic centers, periaqueductal dysfunction gray substances, and dopamine deficiency often leads to chronic pain in TBI [89].

TBI can trigger the development of neurodegenerative diseases due to the formation of neurofibrillary tangle (NFT) and amyloid beta peptide (Aβ) plaques, activation of Aβ-containing microglia, increased levels of α-synuclein (a-Syn), amyloid-beta precursor protein (APP), beta-site amyloid precursor protein cleaving enzyme 1 (BACE1), microtubule-associated protein tau (Tau), apolipoprotein E4 (ApoE4), presenilin-1 (PS1), and caspase-3 [9,10]. In addition, the deposition of hyper phosphorylated Tau in the depth of the sulci and a general lesion of the nervous tissue may underlie the pathogenesis of encephalopathy in TBI [90].

### 4.2. Spinal Cord Injury

Many studies have shown that SCI is often associated with the development of cognitive impairment [6,7,8], encephalopathy [91], depression and anxiety disorders [72,92], epilepsy [75,76], chronic pain [93,94], and neurodegenerative diseases [11,95].

SCI is associated with motor, sensory, and autonomic disturbances below the level of injury, resulting in severe mental and physical suffering. SCI causes hemodynamic disturbances and also affects areas of the brain associated with memory, emotions, and pain regulation as a result of pervasive neurodegenerative processes, which is a powerful inducer of cognitive impairment [96]. SCI can trigger gray matter atrophy in the sensorimotor cortex, cerebellum, medial prefrontal cortex, and anterior cingulate region, increase nerve cell death by triggering apoptotic signaling, and disrupt the neurotransmitter system. This negative front of molecular cellular events caused by SCI can lead to dementia, cognitive impairment, depression and anxiety disorders [97].

SCI increases the risk of developing neurodegenerative diseases. Parkinson’s disease can be caused as a result of neuroinflammation, microgliosis, accumulation of α-Syn and the subsequent loss of dopaminergic neurons in SCI [11]. The main mechanism for the development of neuropathic pain after SCI may be nerve root damage, neuroinflammation, activation of Na^+^ and NMDAR ion channels, and the inhibition of serotonergic, noradrenergic, opioid and gamma-aminobutyric receptors at the area of SCI [98].

### 4.3. Trauma of the Peripheral Nervous System

Peripheral nerve injury can lead to depression. It has been found that damage to peripheral nerves can increase the level of the pro-inflammatory cytokine interleukin-1β (IL-1β) in the frontal cortex and a simultaneous increase in the expression of glial fibrillary acidic protein (GFAP) in the periaqueductal gray (PAG). These negative molecular events may contribute to the development of depression-like behavior [99]. Peripheral nerve injury is reported to induce apoptosis in the dorsal horns of the spinal cord, and trigger mechanisms of excitotoxicity and death of GABAergic interneurons, leading to the development of chronic pain [100].

Of course, in the above sections, we have touched on only part of the large-scale molecular-cellular events during neurotrauma that can lead to certain mental disorders and neurodegenerative diseases. In all these pathological conditions, H_2_S plays an important role; so first, we will consider the main aspects of its endogenous synthesis, catabolism, storage, and a variety of biological effects.

## 5. Metabolism and Functions of H_2_S

### 5.1. Biosynthesis of H_2_S and Its Deposition

The third gasotransmitter, after nitric oxide (NO) and carbon monoxide (CO), is H_2_S. Its discovery as a signaling molecule dates back to 1996, when the endogenous formation of H_2_S in the brain tissue was established with the help of the enzyme cystathionine-β-synthase and its possible role in neuromodulation was assumed. In aqueous solutions, hydrogen sulfide dissociates into H^+^, HS^−^ and S^2−^. Under physiological conditions, approximately 20% of this gas exists in the form of H_2_S, about 80% in the form of HS^−^ and only traces in the form of S^2−^ [101,102].

H_2_S mainly exists as gaseous molecules or sodium bisulfide (NaHS). H_2_S can bind to hemoglobin to form sulfhemoglobin. In addition, proteins containing the iron–sulfur complex and sulfane, which includes hydrosulfides/persulfides, are commonly recognized forms of H_2_S accumulation in the body [31].

**Figure 1:**
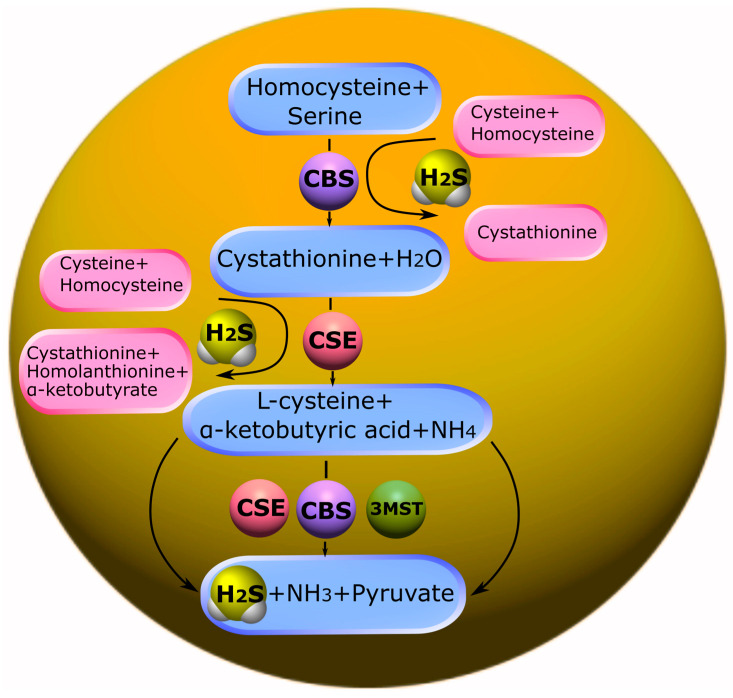
Biosynthesis of H_2_S in the body. Cystathionine-β-synthase (CBS) catalyzes the condensation of homocysteine (Hcy) with serine to form cystathionine, which cleaves cystathionine-γ-lyase (CSE). This results in the synthesis of H_2_S. CBS, CSE and 3-mercaptopyruvate sulfurtransferase (3-MST) catalyze the conversion of cysteine to H_2_S.

The main substrate for the production of H_2_S in humans and animals is L-cysteine, as well as its disulfide form—cysteine. The synthesis of hydrogen sulfide in the body occurs under the influence of the enzymes cystathionine-β-synthase (CBS), cystathionine γ-lyase (CSE) and 3-mercaptopyruvate sulfurtransferase (3-MST) (Figure 1), together with cysteine aminotransferase (CAT) [24,25,101,102].

CBS is predominantly expressed in the brain, liver, kidneys, and pancreas. It is basically a cytosolic enzyme. However, in certain types of cells, it can be localized in the nucleus [103] and mitochondria [104]. CBS expression is controlled by various extracellular and intracellular mechanisms in normal and pathological conditions [105].

CBS catalyzes the condensation of homocysteine (Hcy) with serine to form cystathionine. Subsequently, cystathionine undergoes proteolysis by the enzyme CSE. This leads to the formation of cysteine, which is a precursor of glutathione. It should be noted that in addition to the canonical pathway, CBS is involved in desulfurization reactions that lead to the formation of endogenous H_2_S. The formation of H_2_S may be affected by a thiol-cysteine reaction catalyzed by CBS with the release of s-thiolate. Cysteine may also undergo hydrolysis by CSE to form H_2_S, as well as pyruvate and ammonia. Disruption of the mechanisms of regulation of CBS is associated with a change in the levels of Hcy and/or H_2_S and, inevitably, leads to various pathological conditions (Figure 1) [106,107].

CBS and CSE are jointly involved in the trans-sulfurization pathway, where homocysteine formed in the methionine cycle switches to cysteine synthesis (Figure 1) [108,109,110].

### 5.2. Catabolism of H_2_S

The catabolism of H_2_S has been studied much less than its synthesis in the body. Currently, three pathways of H_2_S catabolism are mainly known: oxidation, methylation and exhalation. The oxidation of H_2_S mainly occurs in hepatocytes. Here, H_2_S is oxidized by mitochondrial enzymes to sulfate with the intermediates persulfide (RSSH), sulfite (SO_3_^2−^), and thiosulfate (S_2_O_3_^2−^) [111,112].

**Figure 2:**
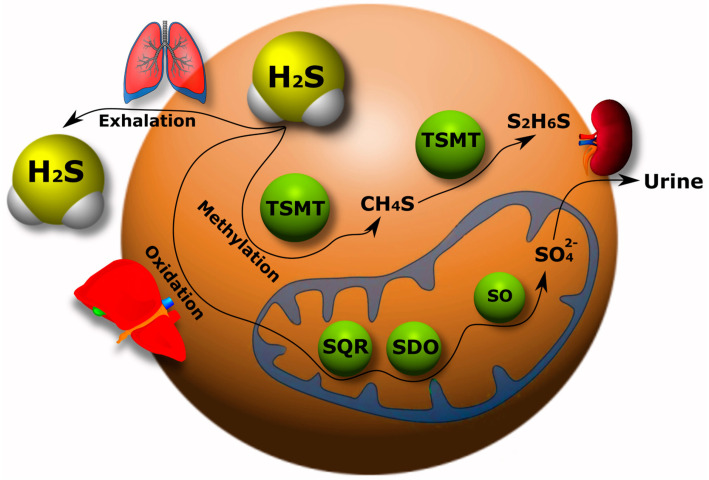
H_2_S catabolism pathways in the body: oxidation, methylation and exhalation. TSMT, thiol-S-methyl transferase; SQR, quinone oxidoreductase; SDO, sulfur deoxygenase; SO, sulfite oxidase.

As a result, most of the H_2_S is excreted in the urine in the form of sulfate. It has been shown that the increase in sulfide oxidation in the kidneys, heart and liver upon administration of exogenous H_2_S is due to an increase in quinone oxidoreductase (SQR) (Figure 2). However, this effect was not observed in the brain tissue, which indicates a defect in the oxidation of sulfides in the nervous tissue of the brain [113].

Free H_2_S exists in low concentration in the blood and decays rapidly. Therefore, it will probably not all be transported to the liver for disposal. The question of H_2_S catabolism in the brain via alternative pathways independent of the liver and kidneys remains open [31].

The methylation of H_2_S occurs primarily in the cytoplasm in contrast to the oxidative catabolic pathway of this gasotransmitter. First, H_2_S is methylated to methane thiol, and then it is methylated to a non-toxic dimethyl sulfide by a thiol-S-methyl transferase (TSMT) (Figure 2). The methylation of sulfides has been found to be a significantly slower process than oxidation [114,115]. H_2_S can be excreted from the body through lung tissue (Figure 2) [31,116].

### 5.3. Various Biological Effects of Endogenous H_2_S

**Figure 3:**
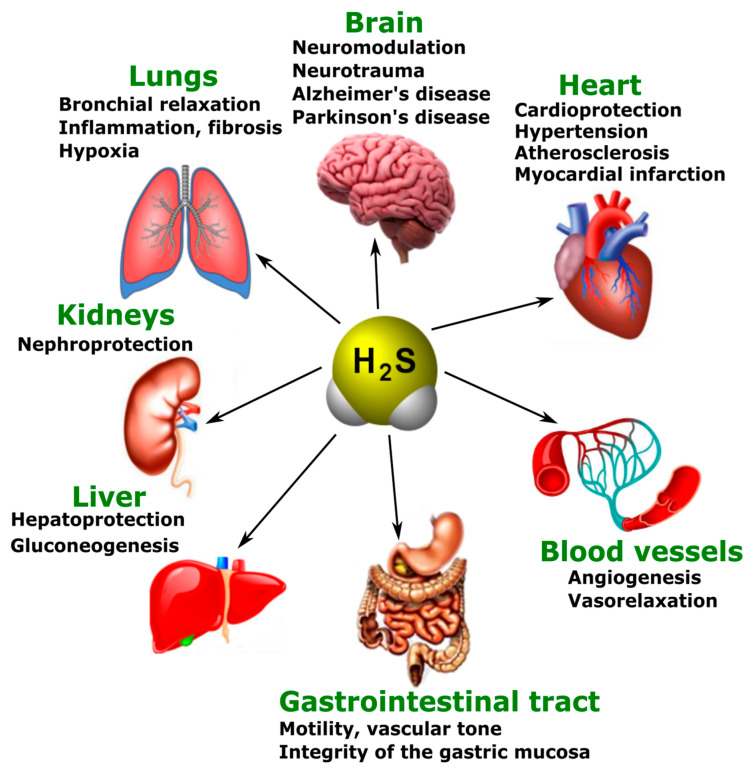
The participation of H_2_S in normal and pathological conditions in the brain, heart, blood vessels, gastrointestinal tract, liver, kidneys, and lungs.

The main physiological effects of H_2_S are neuromodulation, regulation of vascular tone and oxidative stress, anti-inflammatory action, angiogenesis, and energy generation. However, these effects do not exhaust the diversity of the biological actions of H_2_S. Presently, the list of functions performed by this gaseous signaling agent is constantly expanding (Figure 3) [117].

Just like NO, H_2_S is a new generation neurotransmitter that has also been shown to have pronounced neuroprotective effects. In physiological conditions, H_2_S has a role in learning and memory processes. It facilitates long-term potentiation in the hippocampus by activating NMDARs associated with Ca^2+^ channels [118,119]. Disturbances in the endogenous production and metabolism of H_2_S have been observed in neurodegenerative diseases, such as Alzheimer’s (AD) and Parkinson’s disease (PD) [120,121]. Moreover, the use of exogenous H_2_S in these diseases has been proven to have a positive therapeutic effect [122,123].

Researches have shown that exogenous H_2_S reduces early brain injury in subarachnoid bleeding [124], increases motor function, and reduces cortical lesions after traumatic injury [67].

However, the biological effects of H_2_S are far from ambiguous. It has been proven that the neuroprotective effect of H_2_S is due to anti-oxidant, anti-inflammatory and anti-apoptotic properties [32]. However, under certain conditions, H_2_S can contribute to secondary damage to neurons, causing them to be overloaded with Ca^2+^ ions. In experiments on mice, it was found that inhibitors of enzymes for the synthesis of H_2_S can reduce early damage to the blood-brain barrier (BBB) after transient focal cerebral ischemia [125].

Now that we have considered the metabolism of H_2_S, as well as the molecular mechanisms of neurotrauma and its relationship with mental and neurodegenerative diseases, we can move on to the role of hydrogen sulfide in cell death in neurotrauma. This section will help us better understand the H_2_S-dependent signaling mechanisms that underlie mental disorders and neurodegenerative diseases.

## 6. Endogenous and Exogenous H_2_S in Neurotrauma

### 6.1. Endogenous H_2_S Levels in Neurotrauma

Recently, several experiments have been developed to study and detect changes in the concentration of H_2_S in neurotrauma in both animal and human models. The concentration of H_2_S has been shown to be a dynamic system. The level of the endogenous expression of H_2_S and CBS in the blood and brain tends to decrease after neurotrauma. Zhang M and colleagues demonstrated that CBS expression was suppressed in the cerebral cortex and hippocampus of mice in TBI. Furthermore, at first it gradually decreased, reaching the minimal values, and then increased. H_2_S demonstrated dynamic changes in TBI, in parallel with the expression of the key enzyme of its synthesis [126]. There was a significant decrease in CBS and CSE in the hypothalamus and brain stem. Furthermore, 3-MST decreased only in the hypothalamus. At the same time, regular intra-abdominal administration of NaHS restored the levels of CBS and CSE, and of 3-MST [127].

Interesting results were obtained on the TBI model in salmon fish, as the number of CBS^+^ cells in the telencephalon significantly increased after injury compared to the control group [128,129]. In a recent study, TBI was shown to significantly reduce H_2_S/CSE levels without significant changes in CBS expression [130]. In a porcine model of acute subdural hematoma, increased levels of CBS and CSE were observed in neurons, vessels, and parenchyma at the base of the cerebral gyri [131].

Changes in the level of H_2_S in the spinal cord have been reported in various neurodegenerative processes [132]. However, there is practically no information concerning changes in the level of hydrogen sulfide in the spinal cord during traumatic exposure. More scientific data is devoted to the study of the effect of hydrogen sulfide donors on the signaling mechanisms of survival and death of neurons and glial cells in SCI.

### 6.2. Exogenous H_2_S: Between Neuroprotection and Neurodegeneration

**Figure 4:**
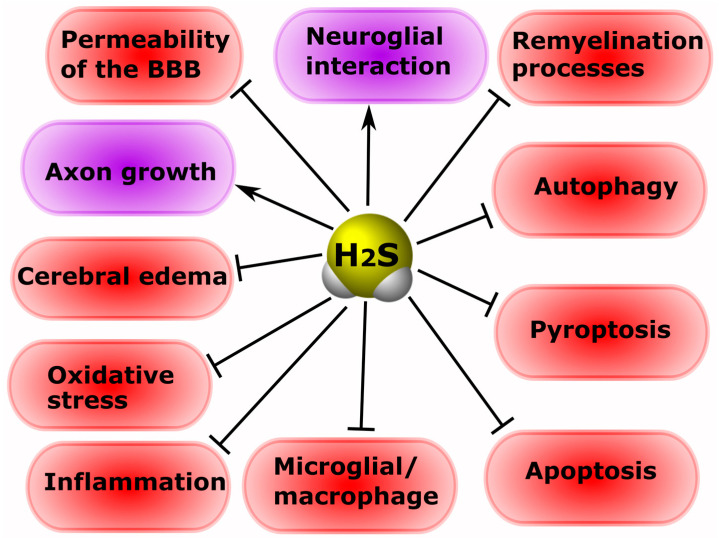
The role of H_2_S in neuroprotection and neurodegeneration in neurotrauma. Arrows with a sharp end—positive regulation; arrows with a blunt end—negative regulation.

It is known that H_2_S is involved in the processes of neuroprotection and neurodegeneration in neurotrauma. The administration of H_2_S donors can protect neurons and prevent the development of hemodynamic disorders in TBI [126]. Increasing the concentration of H_2_S reduces cerebral edema, improves motor activity, and reduces apoptosis and autophagy in an animal model of TBI [133]. According to Jiang and co-authors, H_2_S leads to the activation of antioxidant enzymes, reducing the oxidative damage to nervous tissue cells in TBI [134]. The authors of another study indicate that H_2_S reduces mitochondrial dysfunction and autophagy in TBI (Figure 4) [135].

Increasing the concentration of H_2_S by using a ferrofluid hydrogel (FFH) with iron tetrasulfide (Fe_3_S_4_) significantly reduces activated microglial/macrophage levels and the expression of pro-inflammatory factors, and increases the rate of directional growth of axons in animal models of SCI [136]. The use of H_2_S-releasing silk fibroin hydrogel resulted in a decrease in the level of neuronal pyroptosis induced by TBI [137]. NaHS has been reported to reduce the area of spinal cord infarction in the ischemic-reperfusion model of injury [138]. CBS activation reduces the level of reactive oxygen species, lactate dehydrogenase expression, and apoptosis in the nervous tissue in massive cerebral infarction [139]. H_2_S donors reduce neurological disorders, nerve cell apoptosis, pro-inflammatory secretion, and oxidative stress in spinal injuries [140]. It is also reported that increasing H_2_S levels significantly reduces the permeability of the blood-spinal barrier, improving the recovery of damaged spinal cord neurons [57]. The use of H_2_S-bound nanoparticles promoted the regeneration of damaged spinal cords in rats via the TOR signaling pathway [141]. Intranasal administration of polysulfide prevented neurodegenerative changes in the injured spinal cord [142]. It has been shown that H_2_S is involved in neuroglial interaction in the spinal cord, regulating the survival and death of neurons [143]. This gasotransmitter showed a protective effect against the processes of demyelination in cauda equina fibers in cases of compression injury (Figure 4) [144].

In a mouse model of sciatic nerve damage, H_2_S was shown to significantly reduce neuropathic pain [145]. In addition, CSE and MST have been found to be present in normal nerves, and axotomy activates CSE in Schwann cells [113]. Inhibition of H_2_S production has been reported to improve the growth of regenerating axons and remyelination processes in peripheral nerve injuries (Figure 4) [146].

## 7. The Role of H_2_S in Cell Death in Neurotrauma

### 7.1. Participation of H_2_S in Oxidative Stress

Free radical processes are a vital and important link in metabolism, the violation of which leads to the development of oxidative stress. Violation of the dynamic equilibrium of the oxidant/anti-oxidant system towards free radical oxidation against the background of tension and violation of the coherence of the action of the antioxidant system components leads to the development of oxidative stress [35].

**Figure 5:**
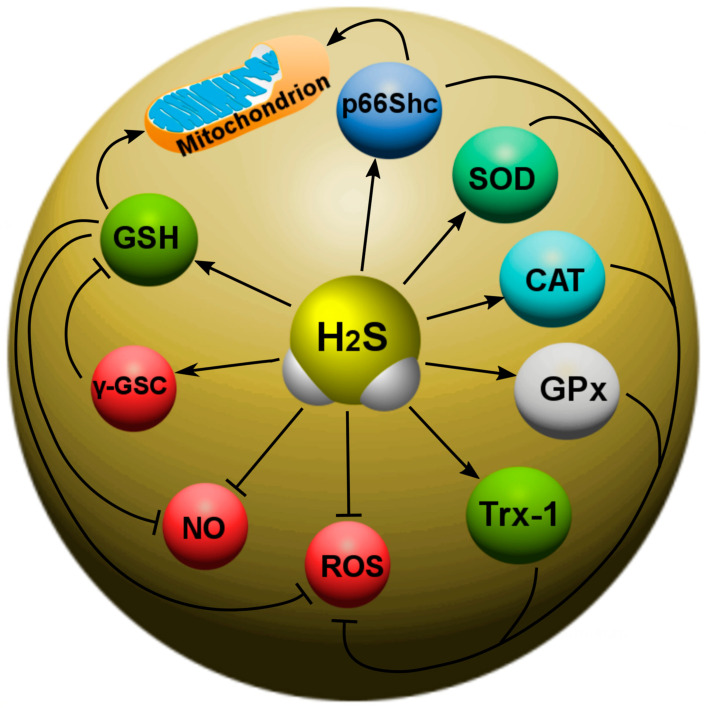
The role of H_2_S in oxidative stress in neurotrauma. H_2_S can directly react with and quench ROS and NO. In addition, H_2_S can increase the level of intracellular reduced glutathione (GSH), which is an antioxidant. However, H_2_S can activate γ-glutamylcysteine synthase (γ-GSC), which limits GSH synthesis. H_2_S is involved in the activation of a number of antioxidant defense enzymes: γ-glutamylcysteine synthase (γ-GSC), thioredoxin (Trx-1), superoxide dismutase (SOD), catalase (CAT), glutathione peroxidase (GPx), and p66Shc.

Recently, the main H_2_S-dependent biological effects in various neurotraumas are considered in the context of the regulation of oxidative stress. It is known that, at 37 °C and a pH of 7.4, more than 80% of H_2_S molecules dissolve in surface waters and dissociate into the ions H^+^, HS^-^ and S^2−^. HS^−^ is a powerful one-electron chemical reagent that effectively traps reactive oxygen species (ROS). Hydrosulfide anions are able to quench ROS by transferring a hydrogen atom or a single electron. The rate of this reaction is directly limited by diffusion. In this case, the reaction of hydrosulfide anions with molecular oxygen proceeds faster in the presence of divalent metal ions. H_2_S effectively interacts with hypochlorous acid (HClO), hydrogen peroxide (H_2_O_2_), lipid hydroperoxides and peroxynitrite (ONOO^−^), neutralizing their oxidative potential [147]. In addition, H_2_S itself is a reducing agent that can directly react and extinguish the superoxide anion (O_2_^−^), NO and its free radical products, as well as other ROS (Figure 5). In addition, H_2_S can act as a trigger molecular agent that triggers antioxidant defense processes [32]. It should be noted that the effectiveness of H_2_S and other simple SH-compounds in neutralizing free radicals is limited due to their low concentration in blood and tissues [148].

In addition, H_2_S can react with NO to form nitroxide (HNO), which is able to bind to the thiol groups of proteins, leading to the formation of disulfide bonds. HNO can modify GSH with the formation of GSH disulfide and sulfinamide, which can increase oxidative stress and inflammatory processes [149].

Studies have shown that H_2_S increases levels of intracellular reduced glutathione (GSH), which is a major antioxidant in the brain [33,34] and spinal cord [150]. Past studies have shown that the H_2_S donor promotes glutamate uptake in astrocytes by enhancing glial glutamate transporter GLT-1 delivery, enhancing cystine transport, and as a result, GSH synthesis [32]. Administration of H_2_S is also associated with elevated levels of GSH in the mitochondria [63]. On the other hand, H_2_S is able to enhance the activity of γ-glutamylcysteine synthase (γ-GSC), which acts as an enzyme that limits the rate of formation of GSH (Figure 5) [25].

Recently, Kimura et al. showed another mechanism of H_2_S effect on the intracellular production of GSH. They reported that the H_2_S produced in cells can be released into the extracellular space and restore cystine to cysteine, which will, thus, be efficiently imported into cells through a cysteine transporter other than the Xc^−^ system and used to synthesize GSH (Figure 5). Meanwhile, Jane et al. also demonstrated that H_2_S increased intracellular GSH production by activating the glutamate-cysteine ligase catalytic subunit (GCLC) and the glutamate-cysteine ligase modifier subunit [32].

H_2_S increases thioredoxin (Trx-1), which is a small (12 kDa) molecule containing the characteristic Cys–Gly–Pro–Cys motif, and the oxidation–reduction of Trx-1 occurs from two of its cysteine residues. Trx-1 is a 12 kDa oxidoreductase enzyme containing a dithiol-disulfide active site that acts as an antioxidant, facilitating the reduction of other proteins by cysteine-thiol-disulfide [69]. Trx-1 has been reported to perform a variety of intracellular and extracellular functions, including capturing ROS and protecting the cell from oxidative stress. Trx-1 reduces hydrogen peroxide with peroxiredoxine (Prx), and oxidized Trx-1 is reduced with thioredoxine reductase. H_2_S has been shown to increase gene transcription and Trx-1 levels [34,69].

H_2_S can bind to the copper (Cu) catalytic center of superoxide dismutase (SOD), which leads to an increase in the rate of absorption of superoxide anions [151]. Recent studies have also shown that H_2_S can attenuate oxidative stress by increasing the activity of catalase (CAT) and glutathione peroxidase (GPx) (Figure 5). In addition, H_2_S can inhibit the mitochondrial production of ROS through p66Shc-dependent signaling. p66Shc is an adaptor protein. It has a negative effect on the ROS-mediated signaling pathway and is involved in the mitochondrial signaling of redox potential. Under oxidative stress, it travels to the mitochondria, binds to cytochrome c (Cyt c), and transfers electrons from Cyt c to molecular oxygen to form ROS. H_2_S interact with p66Shc through sulfhydration and reduces the formation of mitochondrial ROS [35]. However, a high level of H_2_S can, on the contrary, induce the formation of ROS and cause an increase in oxidative stress.

### 7.2. Modulation of the H_2_S Activity of NMDARs and Intracellular Ca^2+^ Homeostasis

H_2_S has been found to modulate NMDAR activity. Protein kinase A (PKA) is known to regulate NMDAR activity. Studies have shown that H_2_S can increase levels of cAMP, which, as a secondary messenger, activates PKA. As a result, the activity of the NMDAR increases. However, H_2_S can activate NMDARs in an independent way. Since NMDARs are extremely sensitive to oxidation and reduction reactions, the biological effects of H_2_S on these receptors may be due to the reduction of disulfide bonds [118]. H_2_S can directly interact with the cysteine residues of receptor subunits, modifying them by S-sulfhydration [119]. It has been established that the hyper activation of the NMDAR, which is an integral part of the pathogenesis of various neurotraumas, leads to Ca^2+^ excitotoxicity and cell death [54,55,118,119].

H_2_S can increase cytosolic Ca^2+^ in neurons by activating slow Ca^2+^ L-type channels. Ca^2+^ L-type channels are one of the main members of the family of potential-controlled calcium channels. The discovery of these channels occurs in response to a strong depolarization of the membrane and causes a prolonged current of Ca^2+^. Ca^2+^ L-type channels are expressed in many tissues, including the nervous system [152]. It is known that these Ca^2+^ channels are involved in the pathogenesis of various injuries of the central nervous system and the PNS [153,154,155]. Thus, it has been shown that H_2_S can increase the Ca^2+^ current in astrocytes, microglia and neurons through the activation of Ca^2+^ L-type channels [156,157]. These H_2_S effects can be significantly reduced by these channel antagonists [158,159]. The role of H_2_S in regulating fast T-type Ca-type CaV 3.2 channels is interesting. Studies have shown that the inhibition of CSE in sciatic nerve injury significantly weakened the activation of CaV3.2 in the ganglia of the posterior roots of the spinal cord. This effect is probably related to the redox H_2_S-dependent modulation of Ca^2+^ channels of this type [119,160]. It is worth noting that the amplification of the Ca^2+^ current through channels of the CaV3.2 type, activated by H_2_S, can participate in the regeneration of neurons [161].

### 7.3. Anti- and Pro-Inflammatory Effects of H_2_S

**Figure 6:**
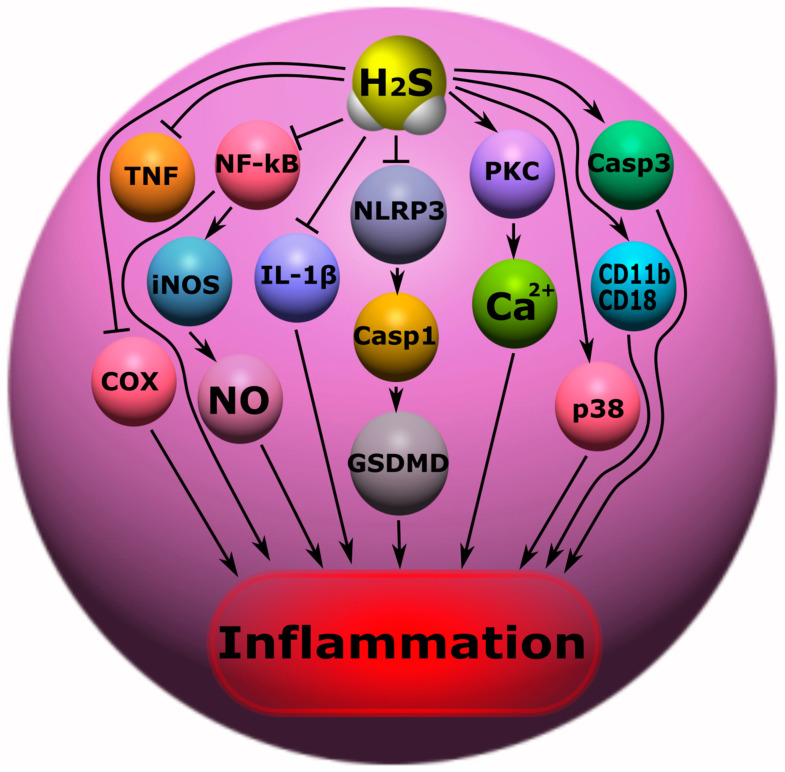
The role of H_2_S in inflammation in neurotrauma. TNF, tumor necrosis factor; COX, cyclooxygenase; NF-κB, nuclear factor kappa-light-chain-enhancer of activated B cells; iNOS, inducible nitric oxide synthase; NO, nitric oxide; IL-1β, interleukin-1 beta; NLRP, nucleotide-binding oligomerization domain, leucine rich repeat and pyrin domain containing; GSDMD, Gasdermin D; Casp1, caspase-1; Casp3, caspase-3; PKC, protein kinase C; Ca^2+^, calcium ions; CaM, calmodulin; p38, p38 mitogen-activated protein kinase; CD11b\CD18, Mac-1β2 integrin; CcO, cytochrome-c-oxidase. Arrows with a sharp end—positive regulation; arrows with a blunt end—negative regulation.

Neuro inflammation is an inflammatory response in the nervous tissue characterized by the activation of glial cells, the involvement of neutrophils and macrophages, and the increased synthesis of cytokines, chemokines, free radicals and secondary messengers. The neuroinflammatory response is characterized by a growing front of molecular cellular events underlying secondary damage to nervous tissue [162,163]. A number of studies have shown that H_2_S plays an important role in inflammatory processes in various pathological conditions, including neurotrauma. The use of ATB-346 (2-(6-methoxynapthalen-2-yl)-propionic acid 4-thiocarbamoyl-phenyl ester), a new H_2_S-releasing derivative of naproxen, in TBI, significantly reduced the inflammatory response, due to the inhibition of oxidative stress, nuclear NF-κB (factor kappa-light-chain-enhancer of activated B cells), leukocyte adhesion to the endothelium, tumor necrosis factor (TNF), and interleukin-1 β (IL-1 β) [67]. It is reported that H_2_S can reduce neuronal inflammation by inhibiting the NLRP3/caspase-1/GSDMD signaling pathway in ischemia/reperfusion brain injury [164]. H_2_S protects retinal ganglion cells in an ischemia/reperfusion injury animal model by regulating a range of signaling proteins involved in inflammation, oxidative stress, and mitochondrial homeostasis (Figure 6) [165].

The family NF-κB is known to consist of transcription factors that play a complex role in immunity and inflammation. NF-κB regulates inflammation through nuclear translocation followed by the expression of pro-inflammatory factors [166]. H_2_S can modulate the activity of NF-κB activity through trans-sulfonation mechanisms, resulting in the inhibition of the nuclear translocation of NF-κB and a reduced inflammatory response [167,168]. In addition, H_2_S can inhibit the phosphorylation of the p65 NF-κB subunit, preventing the activation of this transcription factor [50]. It is worth noting that NF-κB is also a transcription factor for inducible NO synthase (iNOS) [169,170]. iNOS is a Ca^2+^-independent enzyme that generates high levels of NO, in contrast to the constitutive forms of NOS (eNOS/NOS3/endothelial NOS and nNOS/NOS1/neuronal NOS), and is responsible for the increase in oxidative stress and the progression of neuroinflammation [171]. H_2_S can significantly reduce inducible NO production through the inhibition of NF-kB. H_2_S can also inhibit iNOS expression through heme oxygenase (HO-1) activation in macrophages [68]. This gasotransmitter is capable of suppressing the activity of Ca^2+^-dependent NOS [172]. H_2_S has also been shown, in studies, to increase the production of NO in endothelial cells by activating eNOS. In addition, the anti-inflammatory effects of H_2_S in neurotrauma can be realized through the modulation of mitochondrial respiration due to the reversible inhibition of cytochrome-c-oxidase (CcO) (Figure 6) [173]. H_2_S has been reported to reduce inflammation in dorsal root ganglia during sciatic nerve transection [174].

However, the role of H_2_S in inflammation is not so unambiguous. The over production of ROS by neutrophils can cause the oxidation of H_2_S to form sulfite, which leads to leukocyte adhesion and neutrophil activation through the activation of the Mac1 *β* 2 integrin (CD11b/CD18) and protein kinase C (PKC)/Ca^2+^ calmodulin pathway, respectively. In addition, H_2_S can inhibit the breakdown of caspase-3 and the activation of mitogen-activated protein kinase p38 (MAPK) in granulocytes, which is followed by an increase in the inflammatory response (Figure 6) [37,38].

### 7.4. The Effect of H_2_S on the Level of Neurotrophic Factors

Neurotrophic factors are high molecular weight polypeptides that play an important role in the survival, differentiation, and functioning of nerve and glial cells in the brain and spinal cord [175]. Different types of neurotrophic factors, such as nerve growth factor (NGF), brain-derived neurotrophic factor (BDNF), neurotrophin-3 (NT-3), and glial cell-derived neurotrophic factor (GDNF), respectively, play a crucial role in neuronal regeneration in spinal injury, causing the growth of axons and dendrites and showing a neuroprotective effect in TBI neuron models [176,177,178].

It is known that H_2_S is able to modulate the level of neurotrophic factors in normal and pathological conditions [26,179,180]. Thus, in a mouse TBI model, the administration of an H_2_S donor restored GDNF and NGF levels in damaged neural tissue, preserving their neuroprotective effects [67]. The use of a mitochondria-targeted H_2_S donor in middle cerebral artery occlusion has been reported to increase BDNF and NGF expression, reducing ischemic neuronal damage [26]. The administration of NaHS, a donor of H_2_S, increased BDNF levels, probably through activation of the transcription factor cAMP response element-binding protein (CREB), which regulates the gene for this neurotrophic factor in brain damage [181].

H_2_S has also been found to regulate the expression of vascular endothelial growth factor (VEGF), which exhibits neurotrophic and neuroprotective effects in traumatic CNS injury. VEGF is involved in neovascularization, which is necessary for the repair of brain tissue and the regeneration of nerves after neurotrauma. Exogenous H_2_S significantly increased the level of VEGF in the affected area in TBI, which led to the restoration of the BBB [67].

### 7.5. Effects of H_2_S on the Blood-Brain Barrier and Cerebral Edema

Damage to the BBB is the most important pathological substrate of neurotrauma. It was found that H_2_S can participate in the restoration of the functional and anatomical integrity of the BBB [182,183], as well as reduce cerebral edema [25,184]. Thus, in a rat TBI model, it was shown that the use of NaHS, the classic H_2_S donor, reduced the excessive permeability of the BBB by activating the mitochondrial adenosine triphosphate-sensitive potassium channels and reducing oxidative stress. Positive H_2_S-dependent effects may be associated with the inhibition of PKC-α, β I, β II and δ and the activation of PKC-ε, as well as increased levels of Claudin-5, Occludin and ZO-1 [185]. In addition, H_2_S can reduce vascular dysfunction by modulating eNOS levels in TBI [186]. The administration of NaHS significantly reduced damage to the BBB in a spinal cord compression injury model. This H_2_S donor prevented the reduction of the proteins TJ (P120, β-catenin) and AJ (Occlusin), which are among the key components of the BBB [57]. It is known that H_2_S is able to reduce edema in traumatic injury to the naked brain. One of the mechanisms of this H_2_S-dependent effect may be to reduce the destruction of the BBB by suppressing the expression of aquaporin 4 (AQP4) on astrocytes and inhibition of matrix metalloproteinase-9 (MMP-9) [184].

### 7.6. The Role of H_2_S in Remyelination Processes

Remyelination is an important aspect of the recovery of damaged neurons in injuries of the brain [187], spinal cord [188], and peripheral nerves [189]. The processes associated with demyelination develop as a result of secondary damage, leading to a dysfunction of the neuronal network, neurodegeneration, and ultimately, to the death of neurons [188]. It is known that H_2_S can participate in this process [146].

It has been shown that the production of H_2_S in Schwann cells can lead to destruction of the myelin sheath and the recruitment of macrophages. H_2_S positively influences the dedifferentiation and proliferation of Schwann cells in Wallerian degeneration by regulating lysosomal-associated membrane protein 1 (LAMP1), p75 neurotrophin receptor (p75 NTR), c-Jun, and p-ERK1/2. The authors of the study suggest that inhibition of CSE expression may be a potential target in the treatment of pathological processes associated with demyelination [146]. However, there are other studies that show that H_2_S donor treatment leads to a decrease in the demyelination of cauda equina fibers and a decrease in glial cell apoptosis in compression injury [144]. NaHS is reported to promote axonal remyelination and repair by activating the PI3K/AKT/mTOR signaling pathway in TBI [135]. 

### 7.7. H_2_S-Associated Anti- and Pro-Apoptotic Signaling Mechanisms

H_2_S can modulate the apoptosis of neurons and glial cells in neurotrauma. It can act as an anti- and pro-oxidant, as described above, and can regulate the levels of anti- and pro-apoptotic groups of proteins in various traumatic injuries of the nervous system [29,30,31,133]. H_2_S can directly interact with proteins through S-sulfhydration or persulfhydration of cysteine residues on proteins [190], and bind to metalloproteins, modulating their activity and function [191]. In addition, H_2_S can realize its activity through the activation and inhibition of various signaling pathways [25,133].

**Figure 7:**
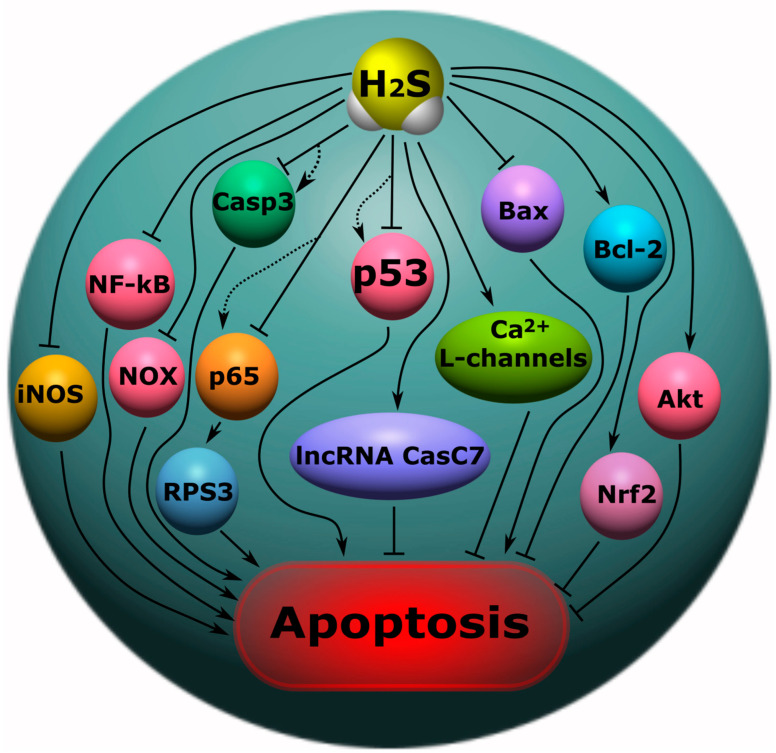
The role of H_2_S in apoptosis in neurotrauma. NF-κB, nuclear factor kappa-light-chain-enhancer of activated B cells; iNOS, inducible nitric oxide synthase; NO, nitric oxide; Casp1, caspase-1; Casp3, caspase-3; NOX, NADPH-oxidase; Bcl-2, B-cell lymphoma 2; Bax, bcl-2-like protein 4; Akt, protein kinase B; p53, tumor protein p53; Nfr2, nuclear factor erythroid 2–related factor 2; p65, RelA; RPS3, ribosomal protein S3; lncRNA CasC7, long non-coding RNA CasC7. Arrows with a sharp end—positive regulation; arrows with a blunt end—negative regulation; dotted line—alternative regulation.

The p53 protein is one of the key pro-apoptotic proteins. It is known as a tumor suppressor and a “guardian of the genome”. This protein controls the most important cellular functions: DNA repair, cell cycle, metabolism, apoptosis, etc. [192,193]. As a transcription factor, p53 is responsible for the expression of many genes, including genes associated with apoptotic cell death [194]. In addition, p53 can participate in transcription-independent processes, regulating mitochondrial functions and triggering the intracellular pathway of apoptosis [195,196]. We have shown the key role of this protein in cell death in neurotrauma in our studies using models of axotomy in vertebrate and invertebrate animals [197,198]. A number of scientific studies have also demonstrated the key role of p53 in the death of neurons and glial cells in various neurotraumas [199,200]. One of the signaling mechanisms for regulating the expression of p53 may be H_2_S (Figure 7). In a recent study, it was shown that H_2_S can inhibit the expression of the p53 protein in damaged neurons, exerting a neuroprotective effect in TBI. Moreover, as the authors suggest, this H_2_S-dependent effect was mediated through the pathway p53/glutaminase 2. The use of an H_2_S donor showed a significant decrease in TUNEL-positive neuronal and glial cells [29]. However, in other studies, H_2_S caused an increase in p53 expression and the initiation of apoptosis [39,40]. Another major pro-apoptotic protein, caspase-3, is also a target for H_2_S. Caspase-3 plays a central role in the cascade of caspases, proteolytic enzymes that sequentially activate each other and underlying proteases [201,202]. H_2_S has been shown to reduce the expression of caspase-3 in damaged neurons and their apoptosis in TBI [29,50]. H_2_S reduced levels of this proapoptotic enzyme in spinal cord injury models (Figure 7) [35,140]. However, there are studies in which H_2_S, on the contrary, induces the expression of caspase-3 and increases apoptotic cell death [203]. H_2_S can affect the activity of caspase-3 by regulating the ROS signaling pathway of activation of this enzyme [29,30]. In addition, the cysteine content of caspase-3 makes it a potential target for direct interaction with H_2_S. This gasotransmitter persulfides caspase-3 using cysteine 163, inhibiting its activity [204]. However, a single concept of the role of the H_2_S-dependent regulation of caspase-3 in survival or cell death still does not exist.

Important proteins involved in the regulation of the permeability of the external mitochondrial membrane and in the regulation of apoptotic signaling are Bcl-2 (B-cell lymphoma 2) and Bax (bcl-2-like protein 4). Bax is known to activate cell death by causing the permeabilization of mitochondrial membranes. The Bcl-2 protein is a molecular antagonist of the proapoptotic effects of Bax. The balance between the levels of these proteins significantly affects cellular fate, leading to either survival or death [205]. A number of studies have shown that H_2_S can act as a modulator of the level of these proteins, regulating the mitochondrial pathway of apoptosis (Figure 7) [29,50]. The anti-apoptotic effect of H_2_S can be realized through the modulation of L-type Ca^2+^ channels and also through a decrease in the level of Bax and caspase-3 in the brain in a model of subarachnoid hemorrhage in rats [206]. It is also reported that H_2_S inhalation in ischemia/reperfusion injury of the brain increases Bcl-2 expression, reduces the level of NF-κB p65, and enhances Akt (protein kinase B) phosphorylation, which may lead to an anti-apoptotic effect. At the same time, exogenous H_2_S suppressed the expression of NOX4 (NADPH-oxidases 4) and CBS (Figure 7) [207].

H_2_S can interact with noncoding RNAs (ncRNAs), which are responsible, in particular, for cell death [208]. It is reported that H_2_S can reduce neuronal apoptosis through the activation of lncRNA CasC7, which has neuroprotective effects, in rat spinal cord ischemia-reperfusion injury (Figure 7) [138].

H_2_S regulates the activity of the NF-κB-dependent signaling pathway, which is a central mechanism in signaling between neurons and glial cells. H_2_S has been shown to reduce NF-kB levels by reducing the expression of iNOS, COX-2 (cyclooxygenases-2), and the cytokines responsible for the inflammatory response (Figure 7) [209,210]. However, H_2_S can sulfhydrate the p65 NF-κB subunit using cysteine-38, which promotes its binding to the coactivator of the ribosomal protein S3 (RPS3) and increases transcriptional activity [24,211]. H_2_S may regulate another transcription factor, Nrf2, associated with anti-apoptotic, anti-inflammatory, and antioxidant effects. H_2_S is reported to reduce nerve cell apoptosis through activation of an Nrf2-dependent signaling pathway in a model of spinal cord injury (Figure 7) [140].

### 7.8. H_2_S-Associated Mechanisms of Autophagy

Autophagy is the main physiological mechanism of intracellular degradation, by which cytoplasmic material is delivered to the lysosome and destroyed in it. It plays a crucial role in maintaining cell survival under various stressors and is a necessary link in the restoration of cellular homeostasis. However, autophagy can lead to cell death [212]. In neurotrauma, the natural process of autophagy is disturbed and can turn into a pathophysiological state, entailing the death of neurons and glial cells [213].

**Figure 8:**
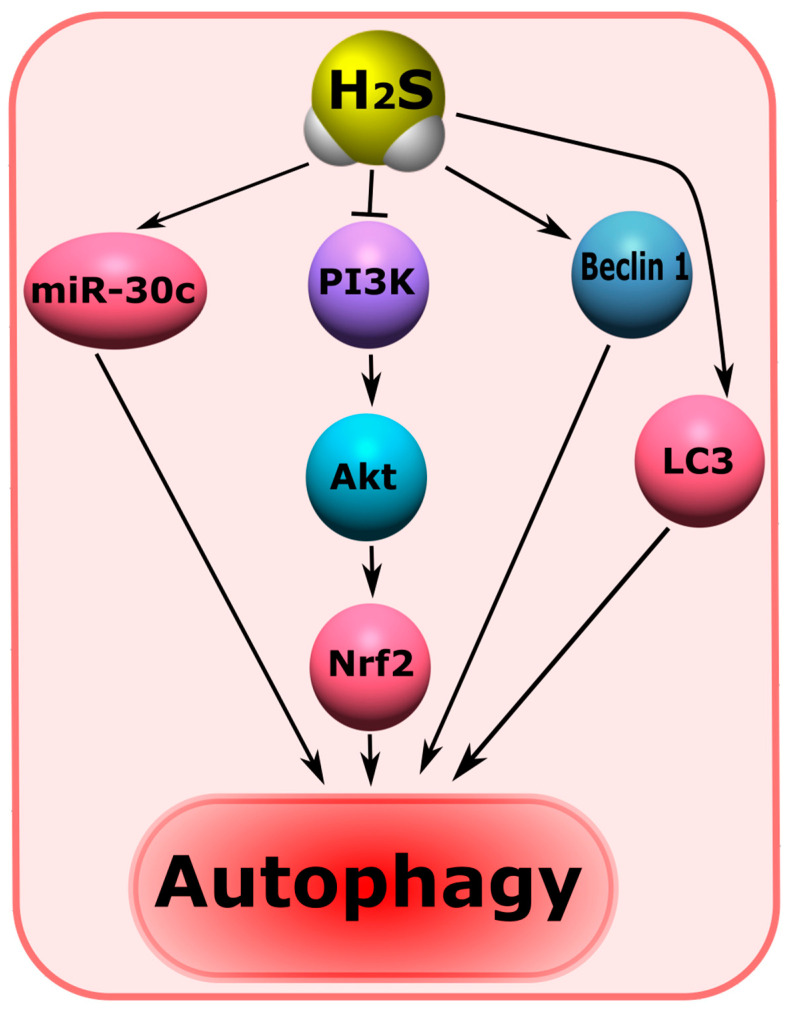
The role of H_2_S in the regulation of autophagy in neurotrauma. miR-30c, micro-RNA 30c; PI3K, phosphoinositide 3-kinase; Akt, protein kinase B; Nfr2, nuclear factor erythroid 2–related factor 2; Beclin, the mammalian orthologue of yeast Atg6; LC3, Microtubule-associated protein 1A/1B-light chain 3. Arrows with a sharp end—positive regulation; arrows with a blunt end—negative regulation.

Autophagy plays an important role in the survival and death of neurons in brain injuries [214]. H_2_S has been shown to regulate autophagy-dependent cell death after TBI [25,50]. One of the mechanisms for blocking autophagic neuronal death in neurotrauma may be the H_2_S-dependent modulation of the PI3K/Akt/Nrf2 pathway and a reduction in oxidative stress [215]. It is known that PI3K/Akt/Nrf2 is a central mechanism involved in autophagy (Figure 8) [216,217].

It is worth noting that autophagy plays a critical role in damage to nerve and glial cells in traumatic and ischemic-reperfusion injuries of the spinal cord [56]. H_2_S may regulate autophagy in spinal cord injuries [57,218]. In a model of ischemic-reperfusion spinal cord injuries, H_2_S has been shown to induce autophagy through the increased expression of miR-30c, Beclin 1, and LC3. miR-30c is a micro-RNA (miRNA) that has been shown to be actively involved in neuroprotection. This miRNA is known to regulate autophagy. In turn, Beclin 1 and LC3 are involved in autophagic processes (Figure 8) [218]. Another study reports that endogenous H_2_S, on the contrary, inhibits autophagy caused by endoplasmic reticulum stress in SCI [57].

### 7.9. H_2_S-Associated Mechanisms of Ferroptosis

Ferroptosis is another type of programmed necrotic cell death, which is characterized by Fe^2+^-dependent lipid peroxidation. It should be noted that, in its morpho-biochemical and molecular genetic mechanisms, ferroptosis differs from apoptosis, autophagy, and necroptosis [219]. In this type of cell death, the size of the mitochondria with condensed dense inner membranes decreases, their cristae undergo changes, up to their complete disappearance, and the mitochondrial membrane ruptures [220]. Traumatic action of the nervous tissue releases a large amount of Fe^2+^, which can lead to ferroptosis of neurons and glial cells [221].

H_2_S plays an important role in ferroptosis. This gasotransmitter can inhibit the process of ferroptosis by increasing the level of antioxidant enzymes, such as GSH, and via ROS uptake [222]. There are practically no data on the functions of H_2_S in the ferroptosis of neurons and glial cells in nervous tissues. For example, H_2_S protects the retinal-blood-brain barrier through the activation of the NRF2/KEAP1 signaling pathway, and via AMPK to p62 phosphorylation [223]. H_2_S is reported to protect BV2 microglial cells by reducing lactate dehydrogenase levels (LDH), oxidative stress, lipid peroxidation, and Fe^2+^ accumulation [224].

### 7.10. H_2_S-Associated Mechanisms of Pyroptosis

Pyroptosis is a type of programmed necrotic cell death that occurs as a result of caspase-1 activation and the disruption of the integrity of the plasma membrane. A feature of this cell death is the active release of IL-1β and IL-18, dependent on caspase-1, which determines the development of an inflammatory reaction [225]. To date, it has been proven that pyroptosis plays an important role in the pathogenesis of injuries of the brain and spinal cord [226]. It has been shown that H_2_S may be a key regulator of the processes associated with pyroptosis [164].

There are not many scientific data on the role of H_2_S in the pyroptosis of neurons and glial cells under conditions of traumatic injury. For example, studies have shown that the use of the new silk hydrogel fibroin (SF), which releases H_2_S, effectively reduces TBI-induced neuronal pyroptosis by inhibiting NOD-, LRR-, and pyrin domain-containing 3 (NLRP3), pyroptosis protein Gasdermin D (GSDMD), caspase-1, and apoptosis-associated speck-like protein containing a CARD (ASC or Picard). In addition, the authors demonstrated that H_2_S inhibits the expression of receptor-interacting serine/threonine-protein kinase 1 (RIPK-1), which is associated with necroptosis [137]. H_2_S may reduce neuronal pyroptosis after severe intracerebral hemorrhage, which often occurs in TBI [227]. NaHS use reduced the pyroptosis of retinal cells and brain neurons through inhibition of the NLRP3/caspase-1/GSDMD signaling pathway in an ischemia/reperfusion injury model [164].

## 8. The Role of H_2_S in Mental Disorders and Neurodegenerative Diseases

### 8.1. Cognitive Impairment

**Figure 9:**
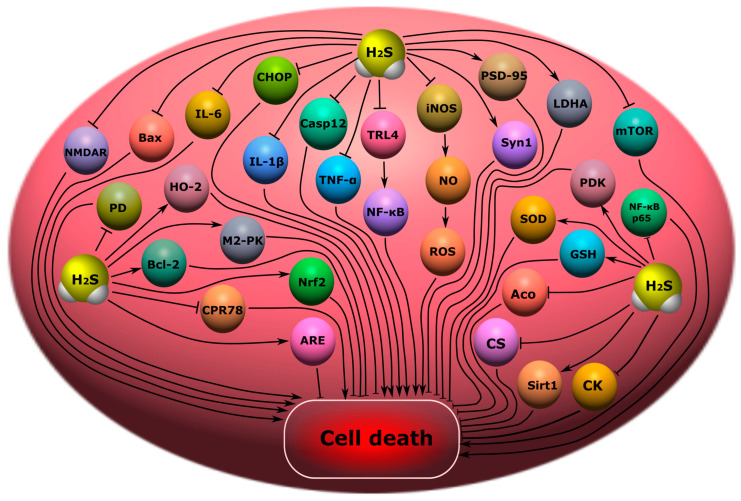
Possible H_2_S-dependent signaling mechanisms that regulate cell death in the nervous tissue in cognitive impairment and encephalopathy. H_2_S, hydrogen sulfide; CHOP, C/EBP homologous protein; Casp12, caspase-12; TRL4, toll like receptor 4; NF-ĸB, nuclear factor kappa-light-chain-enhancer of activated B cells; iNOS, inducible nitric oxide synthase; NO, nitric oxide; ROS, reactive oxygen species; PSD-9, postsynaptic density protein 95; Bax, bcl-2-like protein 4; NMDAR, N-methyl-D-aspartate receptor; IL-6, interleukin-6; IL-1β, interleukin-1β; TNF-α, tumor necrosis factor-α; Syn1, synapsin 1; LDHA, lactate dehydrogenase A; mTOR, mammalian target of rapamycin; PDK, pyruvate dehydrogenase kinase 1; SOD, superoxide dismutase; GSH, glutathione; Aco, aconitase; CS, citrate synthase; Sirt1, NAD-dependent deacetylase sirtuin-1; CK, creatine kinase; NF-ĸB p65, RelA; PD, pyruvate dehydrogenase; Bcl-2, B-cell lymphoma 2; HO-2, heme oxygenase 2; M2-PK, pyruvate kinase M2; CPR78, cuticular protein RR-2 motif 78; Nrf2, nuclear factor erythroid 2–related factor 2; ARE, antioxidant response element.

Endogenous H_2_S may reduce cognitive impairment through the reduction of endoplasmic reticulum stress and the inhibition of caspase-12 and C/EBP homologous protein (CHOP) levels [27]. It has been shown in a rat model of subarachnoid hemorrhage that H_2_S can reduce cognitive deficits by inhibiting the neuroinflammation induced by the TLR4/NF-κB signaling pathway that activates microglial cells (Figure 9) [228].

NaHS, the classic H_2_S donor, had a beneficial effect on the memory of TBI-surviving rats [4]. In a mouse model of surgical trauma accompanied by neuroinflammation, H_2_S improved orientation in the Morris water maze. At the same time, the neuroprotective effect of H_2_S was due to a decrease in the level of pro-inflammatory cytokines TNF-α, IL-1β, and IL-6 in blood serum and in hippocampal cells, which is a key structure of learning and memory [229]. In another study, H_2_S-dependent cytoprotection was associated with a decrease in the level of NO and iNOS expression in hippocampal cells, as well as with the activation of the antioxidant defense system and a decrease in microglial activation (Figure 9) [230].

In a postoperative trauma model, H_2_S reduced cognitive impairment in the Y-maze test; improved the recognition of new objects, and the Morris water maze, by increasing the expression of the synapsin-1 and PSD-95 proteins involved in the process of synaptic plasticity; and prevented a decrease in the density of synapses in the hippocampi of rats. In addition, H_2_S enhanced the Warburg effect, known as aerobic glycolysis, which promotes synaptic plasticity and has a neuroprotective effect [231], in the hippocampal cells of rats with a cognitive deficit against the background of an increase in the expression of hexokinase 2 (HO-2), pyruvate kinase M2 (M2-RK), lactate dehydrogenase A (LDH A), kinase pyruvate dehydrogenase 1 (PDK), increased levels of lactic acid, and reduced expression of pyruvate dehydrogenase (PD) (Figure 9) [232].

H_2_S is reported to improve object recognition and spatial orientation by decreasing Sirt1, oxidative stress, lipid peroxidation, CPR78, CHOP and caspase-12, and increasing GSH and SOD. At the same time, the H_2_S donor significantly reduced the level of apoptosis in the hippocampus, as evidenced by a decrease in TUNEL-positive cells and the Bax\Bcl-2 ratio (Figure 9) [15]. The use of H_2_S donors, ATB-346 and diallyl trisulfide, reduced memory deficits in rats by modulating neuroinflammation, oxidative stress, and the cholinergic system [233].

It is known that damage to the neuromodulatory system often occurs after TBI and underlies cognitive disorders [234]. It is indicated that exogenous H_2_S modulates the level of catecholamines and increases the endogenous synthesis of H_2_S in cognitive impairment [235].

### 8.2. Encephalopathy

H_2_S can significantly attenuate oxidative stress and apoptosis in encephalopathy through activation of the Nrf2/ARE signaling pathway [236]. However, a high content of H_2_S can lead to a decrease in the activity of citrate synthase (CS) and aconitase (Aco) in the mitochondria of neurons of the cerebral cortex, as well as creatine kinase (CK) in this brain structure, the striatum, and the hippocampus in encephalopathy. In addition, H_2_S can enhance lipid peroxidation and disrupt bioenergetic processes in mitochondria [237]. The use of NaHS or S-adenosylmethionine (SAMe), a CBS activator, reduces neuroinflammation and inhibits the expression of pro-inflammatory cytokines (IL-1β, IL-6, TNF-α), restores SIRT1 levels and the phosphorylation of mTOR and NF-κB p65 in neuronal HT-22 cells at high glucose levels, which may indicate H_2_S-dependent neuroprotective defense mechanisms in diabetic encephalopathy [238]. The cytoprotective effect of H_2_S in encephalopathy can be realized through the activation of the antioxidant defense system and a decrease in NMDARs expression (Figure 9) [239].

### 8.3. Depression and Anxiety Disorders

Studies have shown that the administration of NaHS for a week had an antidepressant and anxiolytic effect on mice and rats in various test procedures: forced swimming, tail hanging, and plus maze [240]. In a model of sleep deprivation in rats, H_2_S attenuated depressive and anxiety disorder through the increased expression of Sirt1 in the hippocampus and decreased levels of pro-inflammatory cytokines (IL-1β, IL-6 and TNF-α) and CC motif chemokine ligand 2 (CCL2). At the same time, H_2_S increased the expression of IL-4 and IL-10, which belong to the anti-inflammatory group of cytokines. The use of the Sirt1 inhibitor leveled the neuroprotective effects of H_2_S [241].

**Figure 10:**
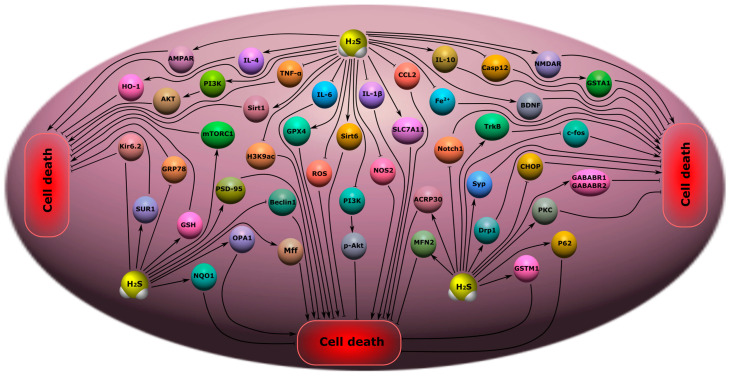
Possible H_2_S-dependent signaling mechanisms that regulate cell death in nervous tissue in depression, anxiety disorders, epilepsy, and chronic pain. H_2_S, hydrogen sulfide; AMPAR, AMPA-type glutamate receptor; IL-4, interleukin-4; IL-6, interleukin-6; IL-1β, interleukin-1β; IL-10, interleukin-10; TNF-α, tumor necrosis factor-α; HO-2, heme oxygenase 2; PI3K, phosphatidylinositol 3-kinase; AKT, RAC-alpha serine/threonine-protein kinase; Sirt1, NAD-dependent deacetylase sirtuin-1; mTORC1, mammalian target of rapamycin complex 1; H3K9ac, acetylated histone H3 lysine 9; GPX4, Glutathione peroxidase 4; Kir6.2, major subunit of the ATP-sensitive K^+^ channel; SUR1, subunit of the ATP-sensitive K^+^ channel; GRP78, glucose-regulated protein 78; GSH, glutathione; PSD-9, postsynaptic density protein 95; NQO1, NAD(P)H quinone dehydrogenase 1; OPA1, optic atrophy 1; Beclin, mammalian orthologue of yeast Atg6; Mff, mitochondrial fission factor; ROS, reactive oxygen species; Sirt6, NAD-dependent deacetylase sirtuin-6; p-Akt, phosphorylated RAC-alpha serine/threonine-protein kinase; NOS2, inducible nitric oxide synthase; SLC7A11, solute carrier family 7 member 11; CCL2, C-C motif ligand 2; Fe^2+^, iron ion; Casp12, caspase-12; NMDAR, N-methyl-D-aspartate receptor; GSTA1, glutathione S-transferase A1; BDNF, brain-derived neurotrophic factor; TrkB, tropomyosin receptor kinase B; c-fos, gene encoding c-fos protein; Notch1, Notch homolog 1; ACRP30, Adipocyte complement-related protein of 30 kDa; MFN2, Mitofusin-2; Syp, synaptophysin; Drp1, dynamin-related protein; CHOP, C\EBP homologous protein; PKC, protein kinase C; P62, sequestosome 1; GSTM1, glutathione s-transferase Mu 1; GABABR1\GABABR2, gamma-aminobutyric acid receptor subunits, GABABR1 and GABABR2.

In another study, the use of NaHS resulted in a reduction in stress-induced depression-like behavior in rats, also via the H_2_S/Sirt1 signaling pathway, which inhibits endoplasmic reticulum stress in hippocampal neurons [242]. H_2_S reduced depressive and anxious behavior in mice by reducing ferroptosis in the H_2_S prefrontal cortex. This H_2_S-dependent effect was due to a decrease in Fe^2+^ deposition, oxidative stress, and an increase in GPX4 and SLC7A11 levels. In addition, the introduction of an H_2_S donor suppressed the activation of microglial cells, reduced the level of pro-inflammatory cytokines, and increased the expression of sirtuin 6 (Sirt6). Moreover, H_2_S increased the deacetylase activity of Sirt6 and reduced the level of acetylated histone H3 lysine 9 (H3K9ac), Notch1, ROS, and the activation of antioxidant enzymes (Figure 10) [243].

Use of the donors H_2_S, llyl isothiocyanate (A-ITC), and phenyl isothiocyanate (P-ITC), reduced depressive behavior by activating the PI3K/p-Akt signaling pathway, as well as reducing oxidative stress, iNOS levels, and inflammatory response in hippocampal cells [244]. In a constraint-induced depression model, H_2_S has been shown to reduce synapse loss and autophagic cell death in the hippocampus via adiponectin activation. NaHS significantly reduced the number of autophagosomes and the level of Beclin 1. This H_2_S donor increased the expression of P62 and adipoxin in the hippocampus of rats with depression-like behavior (Figure 10). The use of Anti-acrp30, which inhibits adiponectin, neutralized the neuroprotective and antidepressant effects of NaHS [245].

It is reported that H_2_S prevents the decrease in the density of dendritic spines and increases the level of mTORC1, as well as the neurotrophic receptors, TrkB, in a rat model of chronic stress-induced depression. As a result of the H_2_S-dependent upregulation of the mTORC1/TrkB signaling pathway, the expression of synaptic proteins, such as PSD-95, synaptophysin, and the AMPA receptor GluR 1/2 subunit, in the hippocampus of rats with depression-like behavior, was increased [246]. H_2_S can realize its antidepressant and anxiolytic effects through the activation of the PI3K/AKT pathway and increased neurogenesis in the hippocampus [247]. Studies have shown that hippocampal levels of H_2_S, CBS, BDNF and PSD-95 are significantly reduced in chronic stress-induced depression in mice. However, the use of S-adenosylmethionine (SAM), which activates CBS, prevented these negative effects and improved the synapse ultrastructure [248]. H_2_S may reduce depressive symptoms through the inhibition of endoplasmic reticulum stress and lower levels of glucose-regulated protein 78 (GRP78), CCAAT/enhancer binding protein homologous protein (CHOP), and caspase-12, in rat hippocampal cells (Figure 10) [249].

### 8.4. Epilepsy

In a mouse model of electrically stimulated epileptic seizures, it has been shown that acute and recurrent seizures lead to a decrease in plasma H_2_S levels. The authors of the study suggest that H_2_S can be considered as a new candidate for the role of a biomarker of severe epileptic seizures. The level of thiocyanate, which is a product of cyanide metabolism via the trans-sulfonation pathway involving H_2_S, was also strongly reduced in the brain and plasma after convulsions. It is noted that the level of GSH did not change. The study yielded important data on the expression of a number of proteins. An increase in the anti-apoptotic protein optic atrophy 1 (OPA1) was observed, as well as mitochondrial fission factor (Mff), mitofusin 2 (MFN2), and dynamin-1-like protein (Drp1) after epileptic seizures (Figure 10) [250].

Studies have shown that a new H_2_S donor based on carbazole has an anti-convulsing effect. In addition, it reduced the level of aquaporin 4, 1β, IL-6 and TNF-α and increased the expression of protein kinase C (PKC). The use of a PKC inhibitor significantly attenuated these H_2_S-dependent effects. The authors suggest that the neuroprotective H_2_S signaling pathway in epilepsy may be mediated through PKC [251]. In addition, H_2_S synthesized on the basis of carbazole from aldehydes suppressed convulsions and increased the expression of the subunits of ATP-sensitive K^+^ channels, Kir6.2 and SUR1 [251]. In a febrile seizure model in rats, NaHS administration protected hippocampal cells, reduced c-fos expression, and increased the expression of gamma-aminobutyric acid receptor subunits, namely GABABR1 and GABABR2 [252]. The use of NaHS significantly increased the latency period between seizures, and suppressed the production of IL-1β and TNF-α in the hippocampus [253]. The inhibition of CBS with aminooxyacetate significantly increased the severity of seizures in rats with lindane-induced refractory generalized epilepsy (Figure 10) [254].

However, there is evidence that H_2_S may exacerbate seizure-like events in rats with entylenetetrazole-induced epilepsy and pilocarpine. Moreover, epileptic activity was recorded using the patch-clamp method in brain sections. The authors of the study suggest that this negative H_2_S effect was due to the activation of the NMDA and AMPA receptors, as well as the voltage-dependent Na^+^ channels, through signaling pathways associated with H_2_S [255]. The use of an H_2_S-sensitive fluorophore has been shown to increase H_2_S in mild epilepsy and significantly decrease in severe epilepsy due to neuronal damage [256]. H_2_S is reported to be elevated during seizures. The use of a CBS inhibitor reduced functional brain hyperemia in epilepsy. NaHS blocked ROS and reduced the cerebrovascular dysfunction caused by epileptic seizures (Figure 10) [257].

### 8.5. Chronic Pain

H_2_S inhalation can reduce neuropathic pain caused by sciatic nerve injury by reducing the expression of pro-inflammatory cytokines and the activation of IL-6-induced microglia in the spinal cord [145]. Using diallyl disulfide (DADS) and morpholin-4-ium 4-methoxyphenyl (morpholino), phosphinodithioate dichloromethane complex (GYY 4137), a sustained release of H_2_S, had an analgesic effect on chronic pain in mice by reducing oxidative stress and apoptosis in the amygdala and lowering the levels of phosphoinositide 3-kinase (PI3K) in this brain structure, gray matter, and the frontal part of the cingulate cortex. In addition, an H_2_S-dependent effect was manifested in the suppression of PKB activation in the infralimbic cortex of the cerebral hemispheres and in the amygdala [258]. Studies have shown that DADS increases the expression of Nrf2, HO-1, NQO1 and GSTM1 in dorsal root ganglia and the periaqueductal gray matter of the cerebral cortex in chronic pain (Figure 10). The authors suggest that H_2_S-induced activation of the antioxidant system in the CNS and PNS contributes to the reduction of pain sensations [259].

Introduction of the slowly releasing H_2_S donors, allyl isothiocyanate (A-ITC) and phenyl isothiocyanate (P-ITC), effectively reduced neuropathic pain, increased HO-1, GSTM1 and GSTA1 levels, and decreased neuroinflammation in the hippocampus and prefrontal brain [260]. NaHS also decreased hyperalgesia and allodynia through activation of the Nrf2/HO-1 signal pathways and decreased TNF-α, IL-1 β, IL-6 and high-mobility group protein B1 (HMGB1) in the dorsal brains of rats with neuropathic pain caused by compression of the ischial nerve (Figure 10) [261]. New σ 1 receptor antagonists, linked to H_2_S donors, have been developed and shown to be effective in pain relief [262].

## 9. Neurodegenerative Diseases

### 9.1. Alzheimer’s Disease

Studies have shown that there is a significant decrease in the level of H_2_S in the brain of patients suffering from AD [28]. Violation of H_2_S-homeostasis towards a decrease in the level of H_2_S is also observed in neurotrauma [126], which indicates the general mechanisms of dysfunction of the H_2_S-synthesizing system in these pathological conditions. H_2_S and its metabolites have been proposed as markers of cognitive impairment and vascular dysfunction in AD [120].

**Figure 11:**
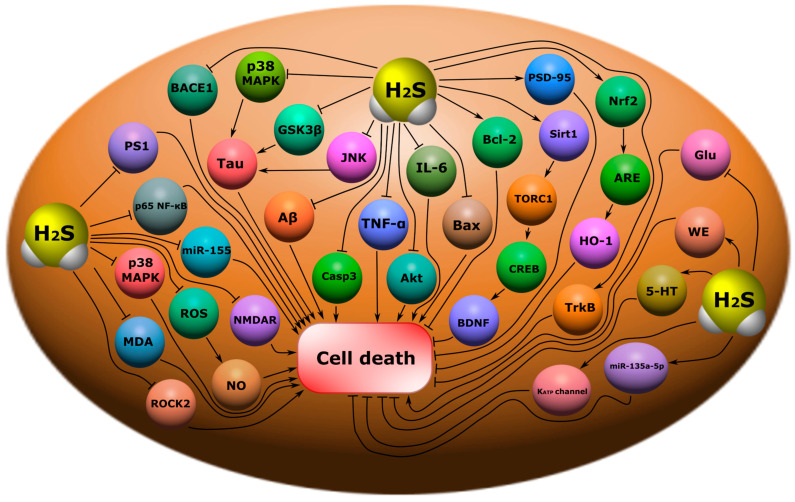
Possible H_2_S-dependent signaling mechanisms that regulate cell death in nervous tissue in neurodegenerative diseases. BACE1, beta-site amyloid precursor protein cleaving enzyme 1; p38 MAPK, p38 mitogen-activated protein kinase; Tau, microtubule-associated protein tau; GSK3β, glycogen synthase kinase-3 beta; JNK, c-Jun N-terminal kinase; Aβ, amyloid beta; Casp3, caspase-3; TNF-α, tumor necrosis factor-α; Akt, RAC-alpha serine/threonine-protein kinase; IL-6, interleukin-6; Bax, bcl-2-like protein 4; Bcl-2, B-cell lymphoma 2; PSD-95, postsynaptic density protein 95; Sirt1, NAD-dependent deacetylase sirtuin-1; TORC1, target of rapamycin kinase complex 1; CREB, cAMP response element-binding protein; BDNF, brain-derived neurotrophic factor; Nrf2, nuclear factor erythroid 2–related factor 2; ARE, antioxidant response element; HO-1, heme oxygenase 1; TrkB, tropomyosin receptor kinase B; Glu, glutamic acid; WE, Warburg effect; 5-HT, serotonin; miR-133a-5p, microRNA 133a-5p; K_ATP_ channel, ATP-sensitive K^+^ channel; PS1, presenilin-1; p65 NF-ĸB, nuclear factor kappa-light-chain-enhancer of activated B cells; miR-155, microRNA 155; ROS, reactive oxygen species; NO, nitric oxide; MDA, malonic dialdehyde; NMDAR, N-methyl-D-aspartate receptor; ROCK2, Rho associated coiled-coil containing protein kinase 2.

H_2_S is reported to inhibit the hyperphosphorylation of Tau by sulfhydration glycogen synthase kinase 3β (GSK3β), improving the motor and cognitive impairment caused by AD [41]. In a triple transgenic mouse model of AD (3×Tg-AD) demonstrating both Aβ and Tau disorders, the treatment for three months with H_2_S significantly protected learning and memory in 3×Tg-AD mice. At the same time, a decrease in amyloid β-plaques in the cortex and hippocampus was observed. These neuroprotective effects were due to the H_2_S-dependent downregulation of c-Jun N-terminal kinase (JNK), extracellular signal-regulated kinases, and p38, which play a key role in phosphorylation, Tau, inflammatory response, oxidative stress, and Ca^2+^-excitotoxicity (Figure 11) [263].

H_2_S may decrease expression mRNA pro-inflammatory cytokines, TNF-α, IL-6 and IL-1β, and prevent synapse loss in AD by increasing synaptophysin and postsynaptic density protein 95 (PSD-95) [264]. The use of NaHS reduced TNF-α, miR-155, pAkt and inhibited apoptosis in AD-induced rats. The morphological picture in the hippocampus improved under the influence of this H_2_S donor [265]. The neuroprotective effects of H_2_S in AD may be due to a decrease in the level of TNF-α, BAX, caspase-3 and the activation of Bcl-2 [266]. It is reported that H_2_S can reduce disturbances in the blood-brain barrier, cerebral circulation, and also modulate NMDAR and synaptic plasticity in AD (Figure 11) [267].

NaHS administration improved spatial memory and learning while reducing apoptosis, microgliosis, and astrogliosis in the hippocampus. There was an H_2_S-dependent decrease in the level of Aβ_1-40_ phosphorylation of p38 MAPK and p65 NF-κB [268]. In another study, H_2_S showed a neuroprotective effect in AD, which was due to a decrease in the level of Aβ_1-40_ and Aβ_42,_ as well as BACE1 (Beta-site APP-cleaving enzyme 1) and PS1. In addition, exogenous H_2_S led to a significant increase in ADAM 17 (a disintegrin and metalloproteinase domain 17) [268]. H_2_S exerts a protective effect against neurotoxins via sulfhydration of the transcription factors Sirt1 and Nrf2, which positively regulate gene expression for a number of antioxidant enzymes, and NF-ĸB, a key player in the inflammatory response. This leads to the regulation of downstream signaling pathways, such as SIRT1/TORC1/CREB/BDNF, TrkB, and Nrf2/ARE/HO-1, or other pathways. In addition, H_2_S can directly bind neurotoxic agents (Figure 11). Violation of these H_2_S-associated mechanisms may underlie the pathogenesis of AD [269].

### 9.2. Parkinson’s Disease

Abnormal levels of H_2_S and its metabolites have been identified in many studies in PD. Thus, it was shown that the level of endogenous H_2_S significantly decreased in the substantia nigra (SN) in PD, and the use of H_2_S donors contributed to the neuroprotective effect and reduced the death of dopaminergic neurons in this pathological condition [270]. The protective effect of H_2_S has been shown in various PD models in vitro and in vivo [271].

It was shown that the use of the H_2_S donor ACS84, a derivative of the compound L-Dopa, reduced motor dysfunction in mice with induced PD by reducing neuronal loss in the SN, via Nrf-2 activation, and through the increased expression of a number of antioxidant enzymes [272]. In addition, four L-Dopa hybrids associated with different H_2_S releasing compounds demonstrated efficacy in the treatment of PD, which could be due to a significant increase in the intracerebral dopamine and GSH reported in PD rats following administration of these drugs [273]. In addition, H_2_S donors can inhibit the ROS-NO cytotoxic pathway in PD (Figure 11) [121].

The H_2_S-dependent antiparkinsonism effect may be caused by a decrease in excess malonic production dialdehyde (MDA), a product of lipid peroxidation [274]. In a 6-hydroxydopamine (6-OHDA) induced PD model, NaHS significantly attenuated motor asymmetry, increased neuronal survival in the SN, and also increased striatal dopamine levels. However, the use of a blocker of ATP-sensitive K^+^-channels, glibenclamide, caused a decrease in the antiparkinsonian effects of NaHS, which indicates the importance of these channels in the implementation of the neuroprotective effects of H_2_S [274]. It is indicated that H_2_S attenuates cognitive impairment, promotes the polarization of microglia from M1 to M2, and enhances the Warburg effect in the hippocampus of rats with PD [245]. In addition, the neuroprotection of H_2_S may be due to the negative regulation of histone acetylation in PD [275]. H_2_S enhances the long-term potentiation of the hippocampus, inhibits the hyper activation of astrocytes, and reduces damage to the dopamine neurons in the striatum in rats with PD induced using 1-methyl-4-phenyl-1,2,3,6-tetrahydropyridine and probenecid (MPTP/p). In addition, exogenous H_2_S increased the level of serotonin and modulated the level of glutamate and γ-aminobutyric acid in the striatum (Figure 11) [276].

Recent studies have shown that an H_2_S-dependent neuroprotective effect may be mediated through activation of the BDNF-TrkB pathway in a mouse model of PD [277]. H_2_S significantly reduces the expression of Rho-associated protein kinase 2 (ROCK2), associated with neurodegenerative processes, by increasing the level of miR-135a-5p in an animal model of PD. It is known that miR-135a-5p inhibits the translation of ROCK2 mRNA in neurons and has a neuroprotective effect (Figure 11) [278].

## 10. Therapeutic Approaches Using H_2_S as a Neuroprotector

Many experimental animal studies have shown that H_2_S has a beneficial effect on the survival of neurons and glial cells in various pathological conditions, including neurotrauma, and psychiatric and neurodegenerative diseases. However, there is no H_2_S-associated neuroprotector that has undergone clinical trials, yet.

The complexity of the development of this drug lies in the fact that it is necessary to understand the molecular signaling mechanisms associated with H_2_S well, which are realized under the conditions of a particular pathological condition. This is not the end of the problem. The therapeutic effect of H_2_S largely depends on its concentration: a level of H_2_S close to the physiological level exhibits a neuroprotective effect, and a high level of H_2_S can lead to cytotoxicity. Therefore, this drug should release H_2_S at the required concentration for a long time and have a neurocumulative effect in order to accumulate precisely in the nervous tissue and not release H_2_S in non-target organs. Moreover, if there are already good developments on the first point, then the situation is more complicated regarding the second [279].

To date, the main H_2_S donors used in research are sodium sulfide (Na_2_S), calcium sulfide (CaS), and NaHS [280]. Their main drawback is the rapid release of H_2_S, which often leads to conflicting results. However, recent studies have increasingly used slow H_2_S donors, which are able to gradually increase the concentration of H_2_S over a long period of time. These donors include allyl isothiocyanate (A-ITC) and phenyl isothiocyanate (P-ITC) [260], as well as ferrofluid hydrogels (FFH) with iron tetrasulfide (Fe_3_S_4_), which have a beneficial effect on nerve and glial cells in pathological conditions [136].

A new water-soluble molecule that effectively but sustainably releases H_2_S is reported, namely GYY4137 (morpholin-4-ium 4-methoxyphenyl(morpholino) phosphinodithioate). It has been shown that the intraperitoneal administration of GYY4137 reduces the negative manifestations of peripheral neuropathy by reducing the secretion of pro-inflammatory cytokines and the activation of microglia and astrocytes in the spinal cord [281]. In addition, GYY4137 protected retinal ganglion cells from oxidative stress and acute ischemic damage in experimental models of glaucoma [282]. In addition, this H_2_S donor affects immune cells involved in the pathogenesis of multiple sclerosis [283].

However, the situation with slow H_2_S donors is also ambiguous. For example, GYY4137 is reported to be ineffective due to the low release of H_2_S, requiring high concentrations. In this connection, new, long-acting H_2_S donors based on GYY4137, such as AP67 and AP105, have been developed. They exhibit high biological activity [284]. FW1256 could be a promising H_2_S donor; it has demonstrated a good anti-inflammatory effect in a number of studies [285]. In addition, the use of ATB-346 (2-(6-methoxynapthalen-2-yl)-propionic acid 4-thiocarbamoyl-phenyl ester), releasing H_2_S, effectively reduced cell death in TBI and SCI by reducing the inflammatory response and oxidative stress [67]. There are also hybrid H_2_S donors that are capable of releasing not only H_2_S, but also another gasotransmitter. For example, NOSH-aspirin (NBS-1120) releases H_2_S and NO during its metabolism and has a pronounced anti-inflammatory and neuroprotective effect. Researchers suggest that it may become a new candidate for the treatment of neuropathological conditions [286]. Of course, there are a number of H_2_S donors that may have the potential to be new generation H_2_S-associated neuroprotectors.

It is possible to modulate the H_2_S level by using inhibitors of key enzymes of its synthesis. The best known inhibitors of CBS are aminooxyacetic acid (AOAA) and hydroxylamine (NH_2_OH), CSE–PAG (propargylglycine) and β-cyanolanine (BCA) [287], and 3 MST–2-[(4-hydroxy-6-methylpyrimidin-2-yl)sulfanyl]-1-(naphthalen-1-yl)ethan-1-one (HMPSNE) [288]. They can be used in cases where there is a dangerous neurotoxic level of H_2_S. However, it should be noted that the presented inhibitors are non-selective, inhibiting the activity of other enzymatic systems, which undoubtedly presents a big problem for their use in medicine and indicates the need to develop inhibitors directed to this class of enzymes [289].

Thus, neuropathology therapy using H_2_S donors/inhibitors is at the preclinical studies stage. However, even now, we can see the therapeutic potential of H_2_S, which has a wide range of biological effects. Further fundamental and practical research on this gasotransmitter is necessary for the effective treatment of many diseases, including pathological conditions associated with the CNS and PNS.

## 11. Conclusions

Injuries of the central and peripheral nervous system and associated neurodegenerative diseases and mental disorders are one of the main causes of disability and death worldwide after cardiovascular and oncological diseases. H_2_S can be considered as a potential molecular target for neuroprotective effects. Definitely, the positive role of H_2_S, manifested in the reduction of oxidative stress, inflammation, demyelination processes, excitotoxicity, apoptosis, autophagy, ferroptosis, and pyroptosis, prevail over its negative effects in the nervous tissue during traumatic damage to the CNS and PNS. In addition, the use of H_2_S donors effectively reduces the symptoms of mental disorders, such as cognitive impairment, encephalopathy, depression and anxiety disorders, epilepsy, chronic pain, and also inhibits the progression of neurodegenerative diseases (Table 2).

Despite the progress achieved in this area, further research into H_2_S-dependent signaling mechanisms underlying the pathogenesis of neurotrauma, mental disorders, and neurodegenerative diseases is needed. This is of fundamental importance and should expand our knowledge of the H_2_S signaling mechanisms in the survival and death of neurons and glial cells. At the same time, these studies are of practical value and can become the basis for the development of new clinically effective H_2_S-associated neuroprotective drugs of a new generation and optimize the existing tactics for the treatment of the pathological conditions discussed above.

## 12. Main Conclusions

### 12.1. Effects of H_2_S in Neurons and Glial Cells in Injuries of the Central and Peripheral Nervous System

1. H_2_S reduces oxidative stress via uptake of ROS and increased levels of GSH, Trx-1, COD, CAT and GPx, and p66Shcis. However, high levels of H_2_S can induce oxidative stress;
2. H_2_S can modulate NMDAR activity through cAMP\PKA, and H_2_S can directly interact with cysteine residues of NMDAR subunits, modifying them by S-sulfhydration. H_2_S can activate slow L-type Ca^2+^ channels, as well as fast T-type Ca-type CaV 3.2 channels;
3. H_2_S can inhibit inflammation by reducing ROS, NF-κB, leukocyte adhesion to the endothelium, TNF, IL-1β, and NLRP3/caspase-1/GSDMD. However, excessive production of ROS can cause the oxidation of H_2_S to form sulfite, leading to leukocyte adhesion and neurophilic activation. H_2_S may increase inflammation through the inhibition of caspase-3 and the activation of p38 protein kinase;
4. H_2_S can increase the level of the neurotrophic factors GDNF, NGF, BDNF, and VEGF;
5. H_2_S is involved in maintaining the integrity of the blood-brain barrier via inhibition of PKC-α, β I, β II and δ, and activation of PKC-ε and increased levels of Claudin-5, Occlusin and ZO-1, as well as in the suppression of the expression of AQP4 on astrocytes and the inhibition of MMP-9 and NOS level modulation;
6. H_2_S can lead to both the destruction of the myelin sheath and the processes of remyelination and the repair of axons by activating the PI3K/AKT/mTOR signaling pathway;
7. H_2_S can regulate apoptosis by acting as an anti- or pro-oxidant, or by interacting with proteins involved in apoptotic signaling. H_2_S can reduce the expression of p53, caspase-3, Bax, NF-κB p65, NOX4, iNOS, COX-2, and increase Bcl-2, ncRNA CasC7 and the phosphorylation of Akt;
8. H_2_S can reduce autophagy by modeling the PI3K/Akt /Nrf2 and ROS signaling pathways. H_2_S may enhance autophagy by increasing miR-30c, Beclin 1, and LC3 levels;
9. H_2_S can inhibit ferroptosis by reducing the level of ROS, the LDH accumulation of Fe^2+^, and by increasing the antioxidant enzyme GSH, as well as through the activation of NRF2/KEAP1 and AMPK to phosphorylate p62;
10. H_2_S can reduce pyroptosis by inhibiting NOD-, LRR-, NLRP 3, GSDMD, caspase-1, and ASC.

### 12.2. Effects of H_2_S in Neurons and Glial Cells in Mental Disorders:

1. H_2_S may reduce cognitive impairment through the inhibition of endoplasmic reticulum stress, caspase-12, CHOP, and TLR4 /NF-κB; a decrease in the level of TNF-α, IL-1β and IL-6, Sirt1, ROS, LP, CPR78, CHOP, caspase-12, Bax; and an increase in synapsin-1 and PSD-95, Bcl-2, HO-2, M2-RK, LDHA, and PDK in the hippocampus. H_2_S modulates the level of catecholamines and reduces the level of apoptosis;
2. H_2_S may reduce apoptosis in encephalopathy through Nrf2/ARE activation. However, a high content of H_2_S reduces the activity of CS, Aco, and CK, and enhances LP in the brain. H_2_S reduces neuroinflammation through a decrease in the level of IL-1β, IL-6, TNF-α, and also restores the level of SIRT1 and phosphorylation mTOR and NF-κB p65 for encephalopathy;
3. H_2_S has an antidepressant and anxiolytic effect through an increase in the expression of Sirt1, Sirt6, IL-4, and IL-10, the activation of PI3K/p-Akt, and a decrease in the level of IL-1β, IL-6, TNF-α, Fe^2+^ deposition, ROS, NOS2, H3K9ac, Notch1, Beclin 1, and GRP78. H_2_S prevents the loss of dendritic spines and increases the level of mTORC1, TrkB PSD-95, synaptophysin, and the AMPA receptor GluR1/2 subunit in depression and anxiety disorders;
4. H_2_S reduces epileptic seizures. H_2_S reduces the level of aquaporin 4, 1β, IL-6, TNF-α, and c-fos, and increases the expression of PKC, Kir6.2 and SUR1, GABABR1 and GABABR2. However, H_2_S can also lead to seizures via activation of NMDARs and AMPARs;
5. H_2_S can reduce neuropathic pain through the inhibition of the expression of microglial activation, a decrease in the level of apoptosis, PI3K, TNF-α, IL-1β, and IL-6, and increased levels of Nrf2, HO-1, NQO1 and GSTM1.

### 12.3. Effects of H_2_S in Neurons and Glial Cells in Neurodegenerative Diseases:

1. H_2_S may reduce the progression of Alzheimer’s disease. H_2_S inhibits hyperphosphorylation Tau; reduces amyloid β-plaques in the hippocampal cortex, neuroinflammation, oxidative stress, JNK expression, p38, TNF-α, IL-6, IL-1β, miR-155, pAkt, Bax, caspase-3, Aβ1-40, Aβ42, the phosphorylation of p38 MAPK, p65 NF-κB, and BACE1; and increases synaptophysin levels, PSD-95, Bcl-2, Sirt1, and Nrf2;
2. H_2_S may reduce the progression of Parkinson’s disease. H_2_S reduces the death of dopaminergic neurons in the SN; increases the expression of Nrf-2, dopamine, and GSH; activates BDNF/TrkB, and miR-135a-5p; and inhibits ROS/NO, LP, and ROCK2.

## Figures and Tables

**Table 1:** Sourcing strategy for each database, further information can be found in Supplementary Materials.

Database	Search Strategy
PubMed	(hydrogen sulfide OR gasotransmitters OR cystathionine-β-synthase OR cystathionine-γ-lyase OR 3-mercaptopyruvate sulfurtransferase OR hydrogen sulfide donors) AND (neurotrauma OR traumatic brain injury OR spinal cord injury OR peripheral nerve injury OR axotomy OR apoptosis OR autophagy OR ferroptosis OR pyroptosis OR cell death OR axon OR dendrites OR dendritic spines OR cognitive impairment OR depression OR anxiety disorders OR epilepsy OR encephalopathy OR chronic pain OR oxidative stress OR inflammation OR neuroinflammation OR brain OR spinal cord OR neurotrophic factors OR cytokines OR neurodegeneration OR Alzheimer’s disease OR Parkinson’s disease OR protein folding)
Scopus	TITLE-ABS-KEY (“hydrogen sulfide” OR “gasotransmitters”) AND TITLE-ABS-KEY (“brain” OR “spinal cord” OR “nerves” OR “neurotrophic factors” OR “mental disorders” OR “neurodegenerative diseases”)
Web of Science	TOPIC (*hydrogen sulfide* OR *gasotransmitters*) AND TOPIC (*neurotrauma* OR *apoptosis* OR *autophagy* OR *pyroptosis* OR *ferroptosis* OR *axon* OR *dendrites* OR *donors* OR *mental disorders* OR *neurodegenerative diseases*)

*—search by the root of the word; “”—exact keyword; ()—grouping of keywords; “AND”—both words; “OR”—either the first or second word.

**Table 2:** The role of H_2_S in cell death in neurotrauma and associated psychiatric and neurodegenerative diseases.

The Role of H_2_S in Cell Death		
Process	Effects of H_2_S on signaling pathways/molecular targets	Effect
Oxidative stress	Increasing GSH, Trx-1, COD, CAT, GPx, p66Shcis, and ROS uptake	Reducing oxidative stress levels
Ca^2+^-homeostasis	Modulation of NMDAR activity via PKA-dependent and independent pathways; regulation of L-type Ca^2+^ channels and fast T-type Ca-type CaV 3.2 channels	Increase/decrease in intracellular Ca^2+^ levels
Inflammation	Inhibition of ROS, NF-κB, leukocyte endothelial adhesion, TNF, IL-1 β, iNOS, NLRP3/caspase-1/GSDMD, cytochrome c oxidase	Reducing the level of inflammation
	Oxidation of H_2_S to form sulfite leads to leukocyte adhesion and neutrophil activation via CD11b/CD18 and PKC/CaM; inhibition of H_2_S cleavage of caspase-3 and p38 in granulocytes	Increased levels of inflammation
Remyelination	PI3K/AKT/mTOR activation	Remyelination and repair of axons
	Regulation of LAMP1, p75NTR, c-Jun, and p-ERK1/2	Increased dedifferentiation and proliferation of Schwann cells in Wallerian degeneration
Apoptosis	Decreased expression of p53, caspase-3, Bax, NF-κB p65, NOX4, iNOS, COX-2; increased levels of Bcl-2, ncRNA CasC7 and Akt phosphorylation	Reducing the level of apoptosis
Autophagy	Inhibition of the PI3K/Akt/Nrf2 and ROS signaling pathways	Decreased autophagy
	Increasing miR-30c, Beclin 1, and LC3 levels	Increase in autophagy
Ferroptosis	Inhibition of ROS, LDH, and Fe^2+^ accumulation; increased GSH levels, NRF2/KEAP1 and AMPK; activation of p62 phosphorylation	Reducing ferroptosis
Pyroptosis	Inhibition of NOD-, LRR-, and NLRP3, GSDMD, caspase-1, and ASC	Reducing pyroptosis
The role of H_2_S in psychiatric disorders		
Cognitive impairment	Inhibition of endoplasmic reticulum stress, caspase-12, CHOP and TLR4/NF-κB; decrease in the level of TNF-α, IL-1β and IL-6, Sirt1, ROS, LP, CPR78, CHOP, caspase-12, Bax; increase in synapsin-1 and PSD-95, Bcl-2, HO-2, M2-RK, LDHA, PDK in the hippocampus; catecholamine level modulation	Reducing the symptoms of cognitive impairment; improving spatial memory, learning, memorization; reducing the level of apoptosis in the cerebral cortex and hippocampus
Encephalopathy	Nrf2/ARE activation; decrease in IL-1β, IL-6, TNF-α levels; restoration of SIRT1 levels and phosphorylation of mTOR and NF-κB p65	Reducing the symptoms of encephalopathy; reducing the level of neuroinflammation and apoptosis
	Decreased activity of CS, Aco, and CK; increased LP in the brain with a high level of H_2_S	Increased neuroinflammation and apoptosis
Depression and anxiety disorders	Increased expression of Sirt1, Sirt6, IL-4, IL-10, NOS2, PI3K/p-Akt, mTORC1, TrkB PSD-95, synaptophysin, and AMPA receptor GluR1/2 subunit; decreased levels of IL-1β, IL-6, TNF-α; deposition of Fe^2+^, ROS, H3K9ac, Notch1, Beclin 1, GRP78	Antidepressant and anxiolytic effect; improvement of memory, learning ability; reduction of apoptosis; loss of dendritic spines
Epilepsy	Decreased levels of aquaporin 4, 1β, IL-6, TNF-α, c-fos; increased expression of PKC, Kir6.2 and SUR1, GABABR1 and GABABR2	Reducing epileptic seizures
	NMDAR and AMPAR activation	Increase in epileptic seizures
Chronic pain	Decrease in PI3K, TNF-α, IL-1β, IL-6; increase in Nrf2, HO-1, NQO1 and GSTM1	Reduced symptoms of chronic pain, neuroinflammation and apoptosis
The role of H_2_S in neurodegenerative disorders		
Alzheimer’s disease	Inhibition of Tau hyperphosphorylation; expression of JNK, p38, TNF-α, IL-6, IL-1β, miR-155, pAkt, Bax, caspase-3, Aβ1-40, Aβ42; phosphorylation of p38 MAPK, p65 NF-κB, BACE1; increased levels of synaptophysin, PSD-95, Bcl-2, Sirt1, Nrf2	Improving memory, learning; reducing the number of amyloid β-plaques in the hippocampal cortex, the level of neuroinflammation and oxidative stress
Parkinson’s disease	Increased expression of Nrf-2, dopamine, GSH; activation of BDNF/TrkB, miR-135a-5p; inhibition of ROS/NO, LP, ROCK2	Reducing the progression of Parkinson’s disease; decreased death of dopaminergic neurons in the SN; neuroinflammation, oxidative stress

## Data Availability

Not applicable.

## Supplementary Materials

The following supporting information can be downloaded at: https://susy.mdpi.com/user/manuscripts/displayFile/120f7af664d45b2bd31fba7da53b59a5/supplementary.
